# Supplementary material for: Mitochondrial Ribosomal Protein MRPS15 Is a Component of Cytosolic Ribosomes and Regulates Translation in Stressed Cardiomyocytes
Source: Int J Mol Sci. 2024 Mar 13;25(6):3250. doi: 10.3390/ijms25063250 (PMC10970015; doi:10.3390/ijms25063250)
Supplement: Supplementary file 1 [file ijms-25-03250-s001.zip › Supplementary Material/David et al Figure S1 (ext data Fig. 4).pptx]

## Slide 1
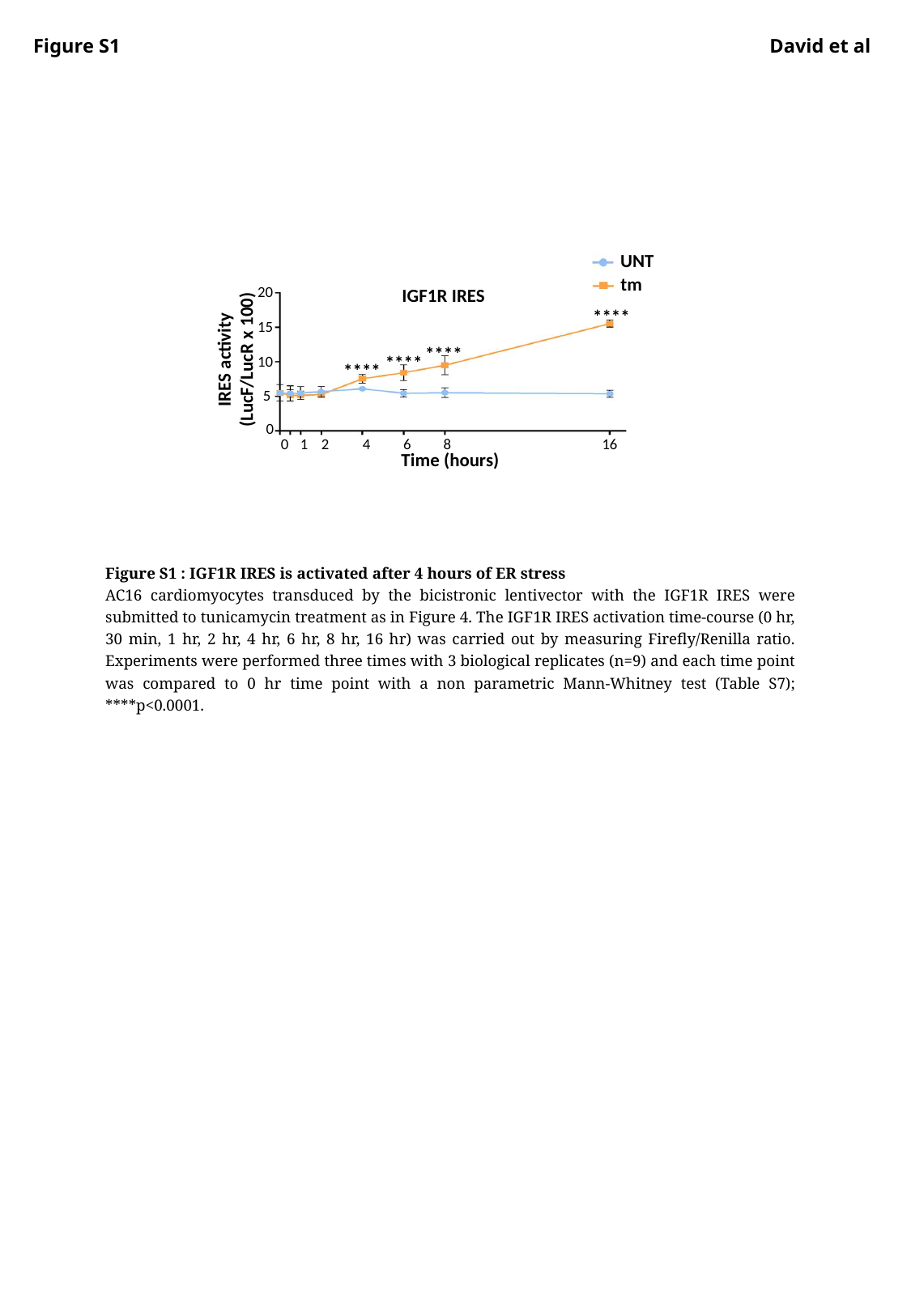

David et al
Figure S1
UNT
tm
20
IGF1R IRES
****
15
IRES activity (LucF/LucR x 100)
****
****
10
****
5
0
1
2
4
6
8
16
0
Time (hours)
Figure S1 : IGF1R IRES is activated after 4 hours of ER stress
AC16 cardiomyocytes transduced by the bicistronic lentivector with the IGF1R IRES were submitted to tunicamycin treatment as in Figure 4. The IGF1R IRES activation time-course (0 hr, 30 min, 1 hr, 2 hr, 4 hr, 6 hr, 8 hr, 16 hr) was carried out by measuring Firefly/Renilla ratio. Experiments were performed three times with 3 biological replicates (n=9) and each time point was compared to 0 hr time point with a non parametric Mann-Whitney test (Table S7); ****p<0.0001.
