# Supplementary material for: Mitochondrial Ribosomal Protein MRPS15 Is a Component of Cytosolic Ribosomes and Regulates Translation in Stressed Cardiomyocytes
Source: Int J Mol Sci. 2024 Mar 13;25(6):3250. doi: 10.3390/ijms25063250 (PMC10970015; doi:10.3390/ijms25063250)
Supplement: Supplementary file 1 [file ijms-25-03250-s001.zip › Supplementary Material/David et al, Figure S2 (ext data 1 Fig. 8).pdf]

Figure S2

DAVID et al.

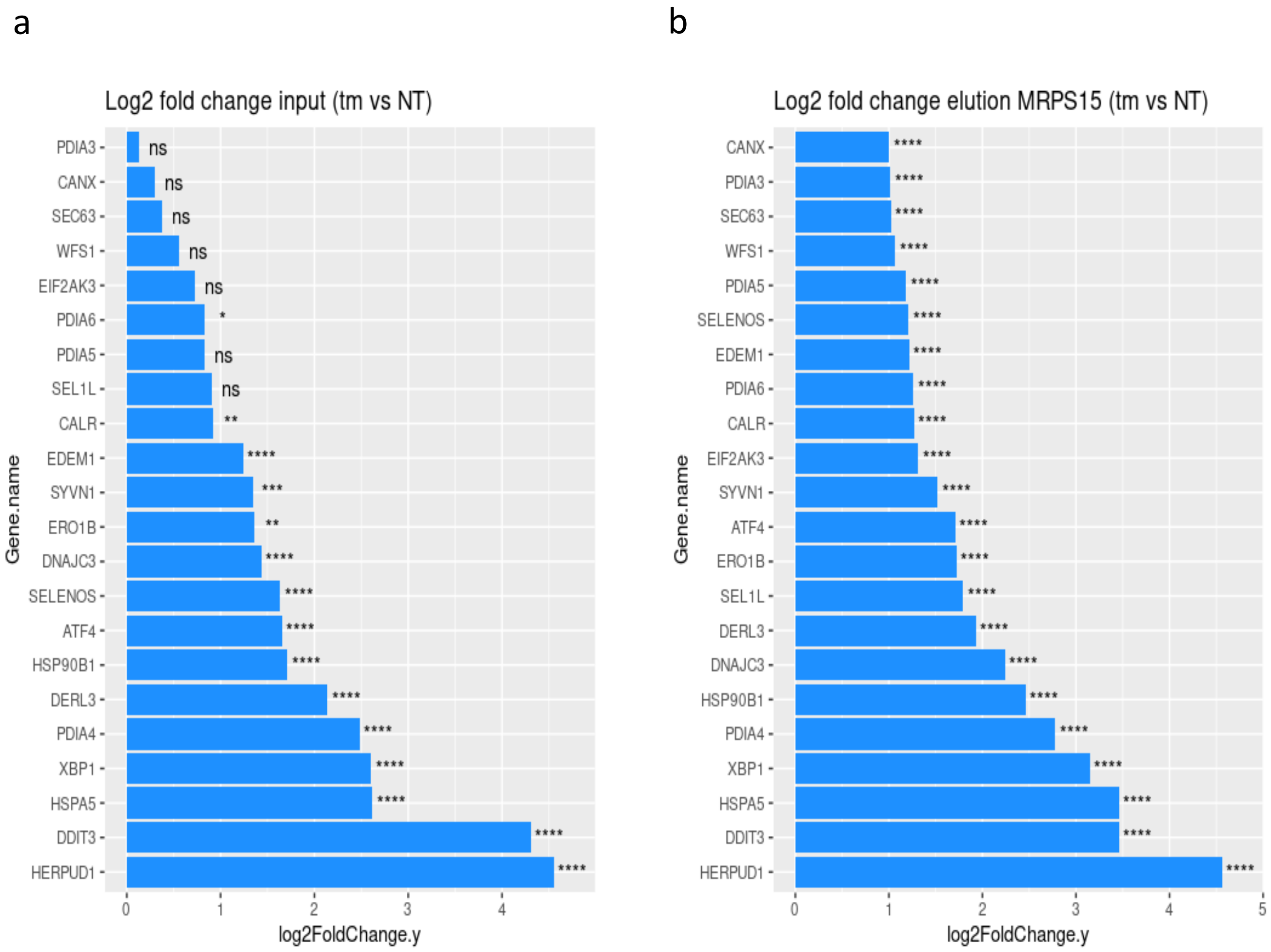

Figure S2 : mRNA enrichment in polysomes of tm-treated versus untreated cells, with or without MRPS15 immunoprecipitation.

mRNA fold change of input (a) or MRPS15 elution (b) samples were compared between treated (tm) and untreated (NT) condition and represented in histograms. Data represent at least 3 independent biological replicates for each condition; *p<0.05, **p<0.01, ***p<0.001, ****p<0.0001.
