# Supplementary material for: Mitochondrial Ribosomal Protein MRPS15 Is a Component of Cytosolic Ribosomes and Regulates Translation in Stressed Cardiomyocytes
Source: Int J Mol Sci. 2024 Mar 13;25(6):3250. doi: 10.3390/ijms25063250 (PMC10970015; doi:10.3390/ijms25063250)
Supplement: Supplementary file 1 [file ijms-25-03250-s001.zip › Supplementary Material/David et al, Figure S3(ext data 2 Fig. 8).pdf]

Figure S3

DAVID et al.

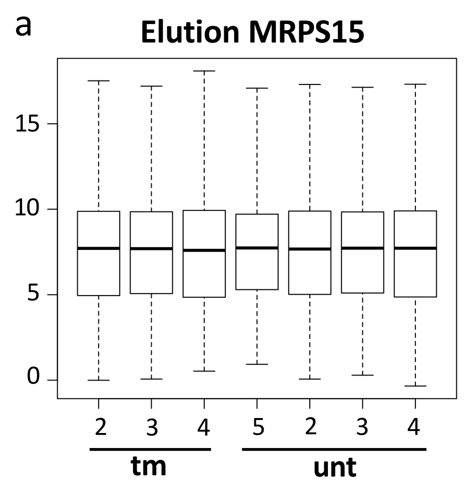

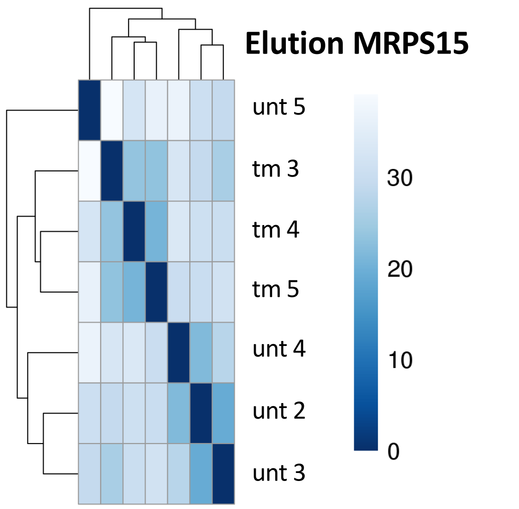

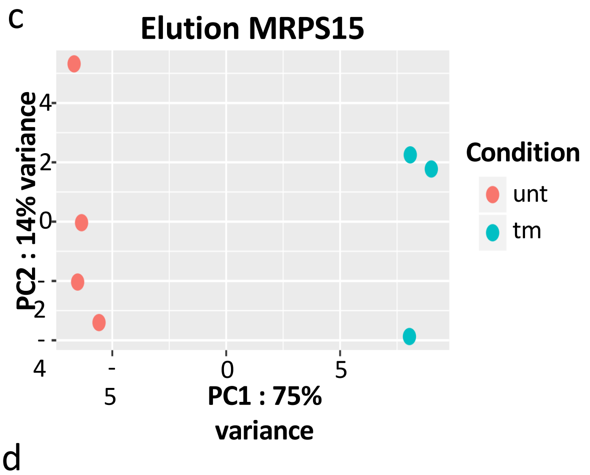

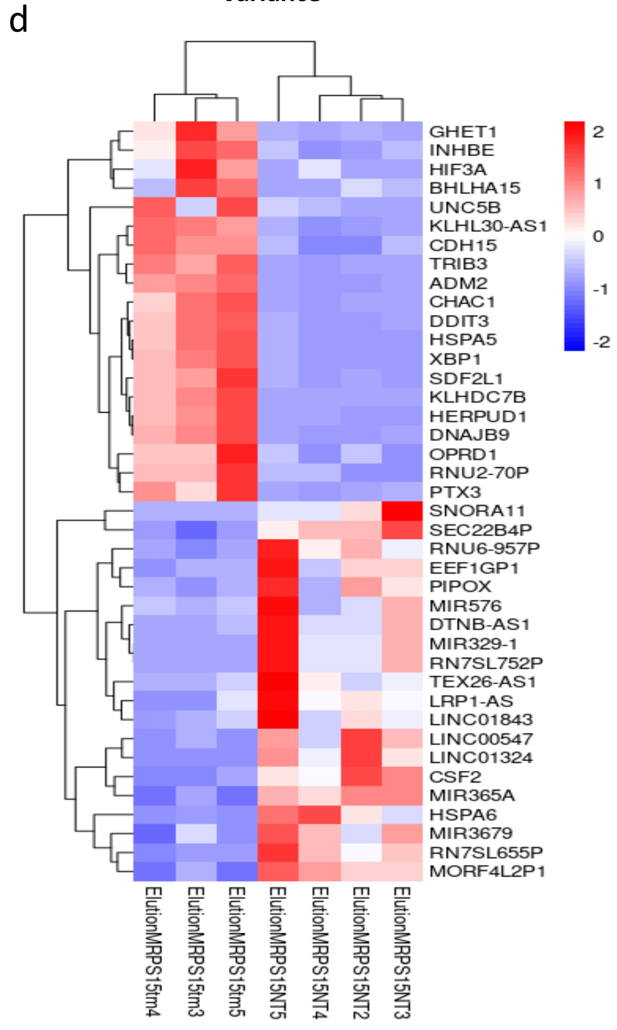

Figure S3 : Quality control analysis and heatmap of MRPS15-IP polysome profiling.

a) Distribution of read counts in libraries of the RNA sequencing were examined before and after normalization. The original read counts were normalized to adjust for various factors such as variations of sequencing yield between samples. These normalized read counts were used to accurately determine differentially expressed genes. Boxplot of the distribution of normalized reads count for genes is shown.

b) Data quality assessments were performed to detect any samples that are not representative of their group, and thus, may affect the quality of the analysis. The overall similarity among samples were assessed by the euclidean distance between samples. This method was used to examine which samples are similar/different to each other and if they fit to the expectation from the experiment design. The shorter the distance, the more closely related the samples are. Samples were then clustered by using the distance and represented in the heatmap.

c) The overall effect of experimental covariates and batch effects. The x-axis is the direction that explains the most variance and the y-axis is the second most. The percentage of the total variance per direction is shown in the label.

d) A bi-clustering heatmap was used to visualize the expression profile of the top 30 differentially expressed genes sorted by their adjusted p-value by plotting their log2 transformed expression values in samples.
