# Supplementary material for: Mitochondrial Ribosomal Protein MRPS15 Is a Component of Cytosolic Ribosomes and Regulates Translation in Stressed Cardiomyocytes
Source: Int J Mol Sci. 2024 Mar 13;25(6):3250. doi: 10.3390/ijms25063250 (PMC10970015; doi:10.3390/ijms25063250)
Supplement: Supplementary file 1 [file ijms-25-03250-s001.zip › Supplementary Material/Tables 22 fev 2024/Table S1 GRP78 eIF2a and P_M.pdf]

a. GRP78 expression (% of T0)

Time	Experiment					Mean	SD
	1	2	3	4	5		
**0 hr**	100.00	100.00	100.00	100.00	100.00	**100.00**	0.00
**0 hr 30 min**	78.94	72.30	78.68	138.63	125.74	**92.14**	31.15
**1 hr**	105.69	121.36	89.18	133.07	65.69	**112.32**	19.07
**2 hr**	94.74	153.69	114.06	98.30	112.02	**115.20**	27.00
**4 hr**	127.44	154.74	112.22	132.49	161.19	**131.72**	17.60
**6 hr**	117.75	152.24	73.64	88.47	160.72	**108.02**	34.71
**8 hr**	154.92	126.35	134.81	209.33	570.30	**156.36**	37.30
**16 hr**	260.39	202.71	197.33	514.03	1328.58	**293.61**	149.69

b. eIF2α phosphorylation (ratio P-eIF2α/total eIF2α)

Time	Experiment					Mean	SD
	1	2	3	4	5		
**0 hr**	1.21	3.29	2.87	1.34	1.06	**2.18**	1.06
**0 hr 30 min**	1.77	1.57	2.34	0.85	1.36	**1.63**	0.61
**1 hr**	2.14	2.79	2.19	1.24	3.10	**2.09**	0.64
**2 hr**	3.88	5.27	7.12	1.50	4.99	**4.44**	2.37
**4 hr**	3.03	6.07	5.40	0.87	7.39	**3.84**	2.37
**6 hr**	4.29	2.91	3.38	1.52	5.31	**3.03**	1.16
**8 hr**	5.76	5.89	4.55	1.55	5.01	**4.44**	2.02
**16 hr**	5.68	4.61	6.05	1.64	10.34	**4.50**	2.00

c. eIF2α phosphorylation (4 hr)

Experiment	Condition	eIF2α (AU)	p-eIF2α (AU)	Ratio	Tm/unt
	unt	654311.7	11974.4	0.018	
**1**	tm	48095.8	23647.4	0.49	**26.87**
	unt	511417.8	12569.6	0.025	
**2**	tm	459455.1	19903.5	0.043	**1.76**
	unt	441216.7	18189.3	0.041	
**3**	tm	519647.2	24571.2	0.047	**1.15**
	unt	712303.7	49168.3	0.069	
**4**	tm	925904.8	51966.9	0.056	**0.81**
	unt	377470.9	19633.8	0.052	
**5**	tm	334187.2	41832.5	0.125	**2.41**

d. GRP78 expression (4 hr)

Experiment	Condition	GRP78 (AU)	GRP78 normalized to Ctrl
	unt	nd	
**1**	tm	21328.4	**nd**
	unt	12912.5	
**2**	tm	13194.6	**1.02**
	unt	36890.2	
**3**	tm	49611.0	**1.34**
	unt	29254.2	
**4**	tm	18791.7	**0.64**
	unt	9921.5	
**5**	tm	6876.5	**0.69**

e. Ratios of polysomes/monosomes (4 hr)

Experiment	Condition	P/ M
	UNT	2.838
**1**	tm	1.914
	UNT	2.395
**2**	tm	1.994
	UNT	2.593
**3**	tm	1.723
	UNT	2.331
**4**	tm	1.637
	UNT	2.300
**5**	tm	1.286

Table S1 : ER-stress marker induction and polysome/monosome ratios.

Kinetics of ER stress induction were performed (5 independent experiments) and ER stress was measured with GRP78 expression (a) and eIF2α phosphorylation (b). 5 independent experiments were performed with a 4 hr treatment to be analysed by mass spectrometry. GRP78 expression (c) and eIF2α phosphorylation (d) and polysome/monosome ratio (e) were measured for each experiment. Mass spectrometry was performed with samples 1, 3, 4 and 5, based on the lower polysome/monosome ratio in the tm condition.
