# Supplementary material for: Mitochondrial Ribosomal Protein MRPS15 Is a Component of Cytosolic Ribosomes and Regulates Translation in Stressed Cardiomyocytes
Source: Int J Mol Sci. 2024 Mar 13;25(6):3250. doi: 10.3390/ijms25063250 (PMC10970015; doi:10.3390/ijms25063250)
Supplement: Supplementary file 1 [file ijms-25-03250-s001.zip › Supplementary Material/Tables 22 fev 2024/Table S3 MRPS15 quantif.pdf]

a. MRPS15 quantification on cytosolic extract

Sample	Experiment				Mean	SD
	1	2	3	4		
**Input unt**	74608.1	115702.5	76231.2	72716.3	**84814.5**	15444.0
**Input tm**	103786.3	98110.3	44773.4	77991.4	**81165.4**	19783.0
**Normalized by unt**						
**Sample**	**Experiment**				**Mean**	**SD**
	1	2	3	4		
**Input unt**	0.88	1.36	0.90	0.86	**1.00**	0.21
**Input tm**	1.22	1.16	0.53	0.92	**0.97**	0.29

b. MRPS15 quantification on cytosolic extract without mitochondria

Sample	Experiment			Mean	SD
	1	2	3		
**Input unt**	233986.1	1708121.8	370668.5	**770925.5**	624797.6
**Input tm**	334023.9	2268352.1	209904.1	**937426.7**	887283.6
**Normalized by unt**					
**Sample**	**Experiment**			**Mean**	**SD**
	1	2	3		
**Input unt**	0.30	2.22	0.48	**1.00**	0.81
**Input tm**	0.43	2.94	0.27	**1.22**	1.15

c. Experiment 1

Fluorescence intensity				Normalized to unt			
unt		tm		unt		tm	
Raw data	Normalized to cell size	Raw data	Normalized to cell size	Raw data	Normalized to cell size	Raw data	Normalized to cell size
422.85	867.50	275.21	760.59	1.71	1.56	1.11	1.37
424.98	917.91	388.25	922.74	1.72	1.65	1.57	1.66
298.36	593.49	465.37	1083.35	1.21	1.07	1.88	1.95
328.45	617.06	432.21	1019.62	1.33	1.11	1.75	1.83
334.90	722.79	235.19	625.37	1.35	1.30	0.95	1.12
250.39	486.74	252.38	627.24	1.01	0.87	1.02	1.13
295.02	607.05	302.79	677.83	1.19	1.09	1.22	1.22
195.90	427.82	317.90	764.83	0.79	0.77	1.29	1.37
207.99	455.17	356.78	874.32	0.84	0.82	1.44	1.57
155.90	381.87	273.52	629.25	0.63	0.69	1.11	1.13
192.86	469.36	226.34	546.54	0.78	0.84	0.92	0.98
215.09	496.54	192.27	464.19	0.87	0.89	0.78	0.83
262.91	580.07	307.48	623.81	1.06	1.04	1.24	1.12
181.17	397.44	325.84	661.58	0.73	0.71	1.32	1.19
219.15	602.49	287.46	710.42	0.89	1.08	1.16	1.28
212.51	459.18	286.74	722.27	0.86	0.82	1.16	1.30
209.76	494.47	286.16	619.71	0.85	0.89	1.16	1.11
280.96	728.33	232.66	483.84	1.14	1.31	0.94	0.87
200.70	441.43	283.44	612.74	0.81	0.79	1.15	1.10
252.20	592.93	305.00	754.96	1.02	1.06	1.23	1.36
267.02	636.76	352.96	777.51	1.08	1.14	1.43	1.40
263.54	599.32	268.12	623.40	1.07	1.08	1.08	1.12
212.14	474.08	252.38	541.46	0.86	0.85	1.02	0.97
283.45	594.43	476.06	1002.82	1.15	1.07	1.93	1.80
304.70	597.46	276.08	649.69	1.23	1.07	1.12	1.17
273.10	580.04	334.26	743.70	1.10	1.04	1.35	1.34
252.51	625.01	376.64	977.02	1.02	1.12	1.52	1.75
290.67	650.01	300.31	622.72	1.18	1.17	1.21	1.12
182.37	417.72	277.35	662.40	0.74	0.75	1.12	1.19
212.40	471.57	255.02	519.29	0.86	0.85	1.03	0.93
228.83	563.65	280.74	635.50	0.93	1.01	1.14	1.14
183.02	405.65	209.32	480.62	0.74	0.73	0.85	0.86
200.13	466.80	152.55	394.58	0.81	0.84	0.62	0.71
254.16	611.24	217.62	509.44	1.03	1.10	0.88	0.91
230.22	603.81	231.70	504.28	0.93	1.08	0.94	0.91
308.52	766.65	243.99	528.59	1.25	1.38	0.99	0.95
178.01	439.79	365.92	847.87	0.72	0.79	1.48	1.52
325.16	690.02	151.57	575.13	1.32	1.24	0.61	1.03
187.13	434.21	246.45	709.65	0.76	0.78	1.00	1.27
265.15	625.02	270.05	654.20	1.07	1.12	1.09	1.17
332.52	697.61	154.86	565.90	1.34	1.25	0.63	1.02

266.02	598.75	317.52	847.18	1.08	1.08	1.28	1.52
366.73	674.03	222.92	482.02	1.48	1.21	0.90	0.87
208.71	464.27	278.08	594.76	0.84	0.83	1.12	1.07
211.25	514.43	337.27	714.59	0.85	0.92	1.36	1.28
186.66	441.67	268.03	582.65	0.75	0.79	1.08	1.05
253.30	625.41	286.87	620.91	1.02	1.12	1.16	1.12
215.86	580.82	191.29	496.78	0.87	1.04	0.77	0.89
183.10	426.73	314.63	705.66	0.74	0.77	1.27	1.27
209.20	477.43	209.75	495.98	0.85	0.86	0.85	0.89
132.65	301.74	221.97	544.09	0.54	0.54	0.90	0.98
		256.82	555.26			1.04	1.00
		197.48	436.71			0.80	0.78
		348.02	1021.58			1.41	1.83
		290.67	703.03			1.18	1.26
		347.25	798.35			1.40	1.43
		308.25	771.78			1.25	1.39
		272.83	684.32			1.10	1.23
		329.25	717.81			1.33	1.29
		260.44	742.06			1.05	1.33
		246.15	586.87			1.00	1.05
		370.03	925.66			1.50	1.66
Mean		Mean		Mean		Mean	
247.26	556.78	283.91	673.21	1.00	1.00	1.15	1.21

d. Experiment 2

**Fluorescence intensity**				**Normalized to unt**			
unt		tm		unt		tm	
Raw data	Normalized to cell size	Raw data	Normalized to cell size	Raw data	Normalized to cell size	Raw data	Normalized to cell size
488.63	968.46	357.19	750.39	1.13	1.06	0.83	0.82
453.43	888.40	275.84	587.79	1.05	0.97	0.64	0.64
410.16	810.96	272.05	580.75	0.95	0.88	0.63	0.63
364.62	791.15	369.85	782.81	0.84	0.86	0.86	0.85
411.38	806.01	283.85	638.86	0.95	0.88	0.66	0.70
394.19	832.58	270.14	558.84	0.91	0.91	0.62	0.61
339.37	726.64	293.53	641.52	0.78	0.79	0.68	0.70
598.75	1121.51	272.02	642.61	1.38	1.22	0.63	0.70
344.55	731.63	169.06	417.86	0.80	0.80	0.39	0.46
385.84	862.84	209.16	477.95	0.89	0.94	0.48	0.52
401.56	898.90	308.36	751.60	0.93	0.98	0.71	0.82
355.47	758.97	262.90	609.94	0.82	0.83	0.61	0.67
320.40	674.85	314.23	609.11	0.74	0.74	0.73	0.66
308.14	689.89	307.49	598.68	0.71	0.75	0.71	0.65
346.69	656.05	376.58	879.40	0.80	0.72	0.87	0.96
385.40	740.86	401.71	997.24	0.89	0.81	0.93	1.09

330.17	662.08	331.17	721.81	0.76	0.72	0.77	0.79
390.02	775.41	419.09	945.99	0.90	0.85	0.97	1.03
513.22	989.58	245.28	645.79	1.19	1.08	0.57	0.70
358.52	775.02	318.42	641.40	0.83	0.85	0.74	0.70
222.28	521.71	336.62	745.05	0.51	0.57	0.78	0.81
340.26	725.79	400.39	823.08	0.79	0.79	0.93	0.90
303.21	672.86	342.76	896.56	0.70	0.73	0.79	0.98
262.99	646.53	315.83	743.75	0.61	0.70	0.73	0.81
393.99	957.38	526.04	980.88	0.91	1.04	1.22	1.07
291.27	707.04	435.13	855.89	0.67	0.77	1.01	0.93
196.01	614.80	340.44	729.80	0.45	0.67	0.79	0.80
347.23	722.21	378.35	800.41	0.80	0.79	0.88	0.87
552.32	1169.48	398.12	1027.02	1.28	1.28	0.92	1.12
479.66	1043.25	374.13	881.68	1.11	1.14	0.87	0.96
311.19	674.72	729.45	1727.79	0.72	0.74	1.69	1.88
300.48	718.10	536.49	1130.03	0.70	0.78	1.24	1.23
340.56	741.82	343.56	746.68	0.79	0.81	0.79	0.81
385.34	792.23	479.89	1078.27	0.89	0.86	1.11	1.18
397.08	852.98	459.01	1066.33	0.92	0.93	1.06	1.16
460.94	1009.81	435.10	1129.56	1.07	1.10	1.01	1.23
322.25	652.97	405.15	868.74	0.75	0.71	0.94	0.95
344.35	829.07	347.14	756.66	0.80	0.90	0.80	0.83
312.50	599.35	451.57	1071.74	0.72	0.65	1.04	1.17
537.89	1010.99	685.51	1459.05	1.24	1.10	1.59	1.59
435.87	951.73	440.54	931.47	1.01	1.04	1.02	1.02
438.68	895.03	421.23	887.07	1.01	0.98	0.97	0.97
367.10	740.82	431.76	889.07	0.85	0.81	1.00	0.97
392.33	832.88	416.09	906.56	0.91	0.91	0.96	0.99
396.58	1071.17	362.80	733.25	0.92	1.17	0.84	0.80
380.90	802.56	370.23	742.40	0.88	0.88	0.86	0.81
599.37	1113.26	380.46	873.12	1.39	1.21	0.88	0.95
636.87	1314.06	334.81	725.68	1.47	1.43	0.77	0.79
597.97	1176.39	301.34	715.88	1.38	1.28	0.70	0.78
497.17	1268.84	392.42	800.32	1.15	1.38	0.91	0.87
534.11	1247.07	547.09	922.61	1.24	1.36	1.27	1.01
408.65	1113.74	656.72	1486.10	0.95	1.21	1.52	1.62
400.89	926.38	415.14	864.01	0.93	1.01	0.96	0.94
418.09	1004.54	282.23	642.60	0.97	1.10	0.65	0.70
484.36	886.33	332.59	724.14	1.12	0.97	0.77	0.79
461.42	882.55	322.20	622.76	1.07	0.96	0.75	0.68
463.90	966.33	521.91	925.18	1.07	1.05	1.21	1.01
398.54	838.97	505.53	1040.50	0.92	0.91	1.17	1.13
726.13	1735.02	353.17	698.85	1.68	1.89	0.82	0.76
472.34	1023.28	328.19	803.81	1.09	1.12	0.76	0.88
917.46	1558.88			2.12	1.70		
576.61	1219.41			1.33	1.33		

626.36	1585.23			1.45	1.73		
903.32	1525.64			2.09	1.66		
564.23	1103.71			1.31	1.20		
Mean		Mean		Mean		Mean	
432.33	917.06	381.58	832.24	1.00	1.00	0.88	0.91

e. Experiment 3

**Fluorescence intensity**				**Normalized to unt**			
unt		tm		unt		tm	
Raw data	Normalized to cell size	Raw data	Normalized to cell size	Raw data	Normalized to cell size	Raw data	Normalized to cell size
353.56	1030.68	714.28	1503.62	0.88	1.22	1.78	1.78
393.64	987.35	539.14	1128.71	0.98	1.17	1.35	1.34
406.21	850.80	440.21	929.00	1.01	1.01	1.10	1.10
379.26	867.35	458.12	888.92	0.95	1.03	1.14	1.05
363.15	692.18	508.17	971.08	0.91	0.82	1.27	1.15
428.23	828.09	534.42	1038.24	1.07	0.98	1.34	1.23
373.47	760.16	481.16	1024.71	0.93	0.90	1.20	1.21
554.31	1119.22	420.22	838.06	1.38	1.32	1.05	0.99
360.69	756.67	463.97	866.17	0.90	0.90	1.16	1.02
327.26	665.10	462.04	958.77	0.82	0.79	1.15	1.13
271.97	891.02	409.13	943.03	0.68	1.05	1.02	1.12
542.09	1056.96	480.46	989.05	1.35	1.25	1.20	1.17
505.14	955.92	452.17	1179.98	1.26	1.13	1.13	1.40
383.84	817.09	576.65	1313.32	0.96	0.97	1.44	1.55
386.87	901.95	624.95	1251.57	0.97	1.07	1.56	1.48
495.36	1022.02	491.20	1232.75	1.24	1.21	1.23	1.46
309.21	644.95	921.00	1786.59	0.77	0.76	2.30	2.11
264.22	522.27	772.95	1737.67	0.66	0.62	1.93	2.06
341.74	689.01	463.49	943.16	0.85	0.82	1.16	1.12
290.26	682.46	824.69	1565.59	0.73	0.81	2.06	1.85
269.43	580.54	554.05	1176.55	0.67	0.69	1.38	1.39
314.18	679.21	567.12	1413.49	0.78	0.80	1.42	1.67
278.55	599.49	440.35	860.16	0.70	0.71	1.10	1.02
334.91	761.40	550.83	1250.41	0.84	0.90	1.38	1.48
485.43	1019.89	710.16	1219.44	1.21	1.21	1.77	1.44
502.30	969.85	511.56	1401.14	1.25	1.15	1.28	1.66
528.69	1082.16	474.36	926.37	1.32	1.28	1.19	1.10
468.25	1083.75	399.08	794.61	1.17	1.28	1.00	0.94
346.66	757.49	528.18	982.81	0.87	0.90	1.32	1.16
464.59	1059.78	483.44	890.73	1.16	1.25	1.21	1.05
536.95	1254.32	342.40	715.88	1.34	1.48	0.86	0.85
630.06	1305.60	466.43	966.35	1.57	1.54	1.17	1.14
515.62	1060.34	469.32	1072.38	1.29	1.25	1.17	1.27
263.96	683.40	650.60	1433.95	0.66	0.81	1.63	1.70

402.99	782.52	500.08	1050.47	1.01	0.93	1.25	1.24
385.20	805.55	517.78	1081.28	0.96	0.95	1.29	1.28
330.08	672.36	502.63	1136.02	0.82	0.80	1.26	1.34
381.38	788.50	465.17	914.33	0.95	0.93	1.16	1.08
526.38	1070.28	603.93	1457.01	1.32	1.27	1.51	1.72
355.01	765.79	501.27	931.49	0.89	0.91	1.25	1.10
356.95	723.36	542.02	1022.64	0.89	0.86	1.35	1.21
377.01	735.95	389.53	812.22	0.94	0.87	0.97	0.96
418.51	830.58	456.08	956.89	1.05	0.98	1.14	1.13
381.63	938.97	512.02	1056.44	0.95	1.11	1.28	1.25
429.54	887.24	633.45	1072.11	1.07	1.05	1.58	1.27
320.91	794.02	382.24	893.80	0.80	0.94	0.95	1.06
395.34	816.00	513.17	1199.91	0.99	0.97	1.28	1.42
324.84	646.26	474.56	927.67	0.81	0.76	1.19	1.10
250.87	511.86	561.75	1227.65	0.63	0.61	1.40	1.45
393.20	760.37	380.45	793.99	0.98	0.90	0.95	0.94
421.65	906.70	469.07	1093.12	1.05	1.07	1.17	1.29
391.17	751.14	447.03	1140.52	0.98	0.89	1.12	1.35
399.31	808.28	388.48	947.06	1.00	0.96	0.97	1.12
327.47	677.23	462.10	931.77	0.82	0.80	1.15	1.10
392.41	744.08	512.65	933.03	0.98	0.88	1.28	1.10
333.92	668.44	602.27	1059.12	0.83	0.79	1.50	1.25
553.96	1012.96	506.92	1059.75	1.38	1.20	1.27	1.25
354.31	908.25	668.84	1222.30	0.89	1.07	1.67	1.45
553.34	1028.11	348.32	897.92	1.38	1.22	0.87	1.06
497.30	986.85	728.20	1469.19	1.24	1.17	1.82	1.74
465.62	876.85	518.97	1225.51	1.16	1.04	1.30	1.45
426.31	859.34	496.86	1146.53	1.07	1.02	1.24	1.36
		489.83	1015.86			1.22	1.20
Mean		Mean		Mean		Mean	
400.27	845.10	520.03	1093.17	1.00	1.00	1.30	1.29

Table S3. MRPS15 quantification in cytosol with or without mitochondria.

a) MRPS15 quantification in total cytosolic extract (left panel) or cytosolic extract without mitochondria (right panel) in stressed and unstressed cells was performed using capillary electrophoresis Simple Western (Fig. 3a).

b) MRPS15 immunofluorescence staining in stressed and unstressed cell. Mitochondria were stained using Mitotracker and a mask of mitochondria was done using threshold Otsu from ImageJ and was substracted from MRPS15 channel to quantify MRPS15 staining. Quantification was performed on 3 independent experiments with at least 50 cells quantified per condition and MRPS15 staining from stressed cells were compared to unstressed cells with a non parametric Mann-Whitney test (Fig. 3b).
