# Supplementary material for: Mitochondrial Ribosomal Protein MRPS15 Is a Component of Cytosolic Ribosomes and Regulates Translation in Stressed Cardiomyocytes
Source: Int J Mol Sci. 2024 Mar 13;25(6):3250. doi: 10.3390/ijms25063250 (PMC10970015; doi:10.3390/ijms25063250)
Supplement: Supplementary file 1 [file ijms-25-03250-s001.zip › Supplementary Material/Tables 22 fev 2024/Table S4 PLA.pdf]

	unt				tm			
	Image	Number of cells	Number of dots	Dots per cell	Image	Number of cells	Number of dots	Dots per cell
**Experiment 1**	1	7	136	19.4	1	6	332	55.3
	2	5	82	16.4	2	11	323	29.4
	3	8	184	23.0	3	7	284	40.6
	4	4	117	29.3	4	8	373	46.6
	5	9	98	10.9	5	14	484	34.6
	6	6	192	32.0	6	9	168	18.7
	7	9	148	16.4	7	4	300	75.0
	8	5	200	40.0	8	6	484	80.7
	9	6	110	18.3	9	8	368	46.0
	10	6	68	11.3	10	9	991	110.1
	11	6	48	8.0	11	8	407	50.9
	12	11	152	13.8	12	8	283	35.4
		**82**	**1535**			**98**	**4797**	
	**Mean**	**18.7**		**19.91**	**Mean**	**48.9**		**51.9**
	unt				tm			
	Image	Number of cells	Number of dots	Dots per cell	Image	Number of cells	Number of dots	Dots per cell
**Experiment 2**	1	2	108	54.0	7	8	218	27.3
	2	7	134	19.1	8	8	238	29.8
	3	7	257	36.7	9	7	262	37.4
	4	6	185	30.8	10	9	330	36.7
	5	6	283	47.2	11	12	496	41.3
	6	8	310	38.8	12	11	391	35.5
	7	6	364	60.7	13	7	471	67.3
	8	8	313	39.1	14	9	452	50.2
	9	8	226	28.3	15	7	176	25.1
	10	4	138	34.5	16	11	191	17.4
	11	5	177	35.4	17	9	182	20.2
	12	7	178	25.4	18	8	172	21.5
					19	8	244	30.5
		**74**	**2673**			**8.8**	**294.1**	
	**Mean**	**36.1**		**37.5**	**Mean**	**33.5**		**33.9**
	unt				tm			
	Image	Number of cells	Number of dots	Dots per cell	Picture	Number of cells	Number of dots	Dots per cell
**Experiment 3**	1	5	252	50.4	1	5	80	16.0
	2	6	144	24.0	2	7	44	6.3
	3	9	172	19.1	3	4	66	16.5
	4	5	105	21.0	4	5	64	12.8
	5	10	166	16.6	5	8	51	6.4

	6	5	57	11.4	6	5	98	19.6
	7	9	63	7.0	7	7	76	10.9
	8	6	75	12.5	8	8	60	7.5
	9	9	114	12.7	9	3	90	30.0
	10	8	191	23.9	10	5	70	14.0
	11	7	162	23.1	11	6	74	12.3
	12	7	153	21.9	12	6	72	12.0
	13	10	140	14.0	13	7	77	11.0
	14	9	49	5.4	14	6	70	11.7
	15	5	59	11.8	15	6	93	15.5
	**Mean**	**110**	**1654**	**18.3**	**Mean**	**88**	**1085**	**13.5**
		**15.0**				**12.3**		
**Experiment 4**	**unt**				**tm**			
	Image	Number of cells	Number of dots	Dots per cell	Image	Number of cells	Number of dots	Dots per cell
	1	9	108	12.0	1	7	581	83.0
	2	6	162	27.0	2	5	134	26.8
	3	7	143	20.4	3	6	159	26.5
	4	6	202	33.7	4	6	137	22.8
	5	7	175	25.0	5	6	98	16.3
	6	7	88	12.6	6	7	116	16.6
	7	7	166	23.7	7	7	93	13.3
	8	11	178	16.2	8	7	124	17.7
	9	7	171	24.4	9	7	157	22.4
	10	5	143	28.6	10	9	155	17.2
	11	6	114	19.0	11	8	192	24.0
	12	7	114	16.3	12	5	289	57.8
	13	8	176	22.0	13	6	139	23.2
	14	5	187	37.4	14	4	122	30.5
	15	6	214	35.7	15	4	183	45.8
					16	8	205	25.6
					17	8	171	21.4
	**Mean**	**104**	**2341**	**21.6**	**Mean**	**110**	**3055**	**28.9**
		**22.5**				**27.8**		

Table S4. Proximal ligation assay of MRPS15 with RPS7.

Proximal ligation assay using MRPS15 and RPS7 antibody was performed on unstressed or stressed cells to visualize colocalization of MRPS15 with ribosome. For unbiased analysis dots quantification was performed after applying threshold otsu of ImageJ software and quantification done on 4 independent experiments with at least 12 images per condition and dots number of stressed cells condition was compared to unstressed condition using a non parametric Mann-Whitney test (Fig. 3c).
