# Supplementary material for: Mitochondrial Ribosomal Protein MRPS15 Is a Component of Cytosolic Ribosomes and Regulates Translation in Stressed Cardiomyocytes
Source: Int J Mol Sci. 2024 Mar 13;25(6):3250. doi: 10.3390/ijms25063250 (PMC10970015; doi:10.3390/ijms25063250)
Supplement: Supplementary file 1 [file ijms-25-03250-s001.zip › Supplementary Material/Tables 22 fev 2024/Table S5 Co-IP V2 29012024.pdf]

a. Cytosolic fraction, anti-MRPS15

	Sample	Experiment			Mean	SD
		1	2	3		
MRPS15	Input unt	233986.1	1708121.8	370668.5	**770925.7**	624797.6
	Input tm	334023.9	2268352.1	209904.1	**937426.7**	887283.6
	Elution IgG unt	582755.7	4173010.1	799724.1	**1851830**	1547453.4
	Elution IgG tm	447621.5	2862904.9	736728.4	**1349084.9**	1009213.3
	Elution MRPS15 unt	1214224.5	28026113.3	1450148.6	**10230162.1**	11863967.4
	Elution MRPS15 tm	1529598.8	8576962.6	1394634.5	**3833732**	3162153.8

b. Cytosolic fraction, anti-RPS2

	Sample	Experiment			Mean	SD
		1	2	3		
RPS2	Input unt	654978.6	2754395.2	750156.3	**1386510.03**	911923.4
	Input tm	794446.1	4832281.2	3712050.5	**3112925.93**	1545653.2
	Elution IgG unt	2512238.7	10463478.1	11240391.1	**8072035.97**	3706531.5
	Elution IgG tm	1476950.6	11565894.4	9445851.9	**7496232.3**	4012854.5
	Elution MRPS15 unt	4962664.4	6553871.1	11553494.8	**7690010.1**	2575656.5
	Elution MRPS15 tm	2866719.7	10571996.6	3586309.7	**5675008.67**	3264658.6

c. Cytosolic fraction, anti-RPL10A

	Sample	Experiment			Mean	SD
		1	2	3		
RPL10A	Input unt	54594.6	406247.6	55485.9	**172109.4**	156092.2
	Input tm	47972.5	698432.8	264097.6	**336834.3**	241065.7
	Elution IgG unt	760576.9	2874400	1795445.8	**1810140.9**	709506.1
	Elution IgG tm	1966437	1787059.9	1062709.8	**1605402.2**	361795
	Elution MRPS15 unt	561725	2221278	1732357.3	**1505120.1**	628930.1
	Elution MRPS15 tm	474567.9	1452610.8	981694.2	**969624.3**	330037.6

Table S5. Co-immunoprecipitation of ribosomal proteins with MRPS15.

a-c) MRPS15 immunoprecipitation experiment was performed using cytosolic extracts of unstressed or stressed cells without mitochondria. MRPS15 immunoprecipitation and RP co-immunoprecipitation were analyzed using capillary electrophoresis. Experiments were performed 5 times (Fig. 3d), and representative experiments are shown.
