# Supplementary material for: Mitochondrial Ribosomal Protein MRPS15 Is a Component of Cytosolic Ribosomes and Regulates Translation in Stressed Cardiomyocytes
Source: Int J Mol Sci. 2024 Mar 13;25(6):3250. doi: 10.3390/ijms25063250 (PMC10970015; doi:10.3390/ijms25063250)
Supplement: Supplementary file 1 [file ijms-25-03250-s001.zip › Supplementary Material/Tables 22 fev 2024/Table S6 Kinetics of FGF1 IRES activity.pdf]

a. LucF activity in untreated condition

Firefly	Experiment	A	B	C	Mean	SD	Mean	SD
	1	330238.0	263198.0	315056.3	302830.8	35152.4		
	2	121658.5	107024.5	118294.8	115659.3	7664.7		
**0 hr**	3	125927.2	116417.2	110414.8	117586.4	7822.0	**178692.1**	94910.2
	1	304498.0	306038.5	279580.7	296705.7	14850.7		
	2	131076.2	114052.0	116870.8	120666.3	9124.7		
**30 min**	3	129730.5	114529.0	132961.2	125740.2	9842.7	**181037.4**	87354.3
	1	344261.5	329167.7	306828.3	326752.5	18833.1		
	2	141598.0	133349.5	145837.5	140261.7	6350.3		
**1 hr**	3	124670.5	115309.5	97870.8	112616.9	13601.2	**193210.4**	101585.6
	1	229780.3	265682.7	267559.7	254340.9	21290.8		
	2	110719.2	135648.8	142098.2	129488.7	16571.7		
**2 hr**	3	153142.2	113520.5	113098.2	126586.9	22998.5	**170138.9**	65604.2
	1	294175.7	281601.0	280903.7	285560.1	7469.4		
	2	142235.5	159202.2	138444.8	146627.5	11053.7		
**4 hr**	3	162518.8	124111.5	174824.5	153818.3	26452.4	**195335.3**	69340.9
	1	277832.7	299841.3	266673.3	281449.1	16877.1		
	2	162985.0	152182.3	141060.0	152075.8	10962.9		
**6 hr**	3	86215.3	55599.3	74044.7	71953.1	15414.8	**168492.7**	92419.5
	1	219066.0	204382.3	272146.3	231864.9	35649.0		
	2	184948.3	168322.3	178111.7	177127.4	8356.6		
**8 hr**	3	104806.7	90652.3	111124.7	102194.6	10483.2	**170395.6**	59504.9
	1	260388.0	226391.3	281572.3	256117.2	27837.3		
	2	134752.7	149038.0	146006.0	143265.6	7526.6		
**16 hr**	3	94732.7	109014.0	114183.3	105976.7	10074.8	**168453.1**	69403.1

b. LucR activity in untreated condition

Renilla	Experiment	A	B	C	Mean	SD	Mean	SD
**0 hr**	1	2344036.2	1988893.0	2333051.2	2221993.4	201945.6	**1461994.1**	583372.5
	2	1195947.5	1080045.5	1191256.5	1155749.8	65603.8		
	3	989012.7	1014182.5	1021522.0	1008239.1	17050.1		
**30 min**	1	2383044.5	2379841.5	2266075.5	2342987.2	66626.7	**1565884.5**	589103.9
	2	1268536.2	1203962.5	1282526.2	1251674.9	41908.1		
	3	1174022.3	1013948.5	1121003.7	1102991.5	81542.9		
**1 hr**	1	2924972.0	2688764.5	2472769.8	2695502.1	226176.4	**1791444.7**	705166.3
	2	1473335.0	1483665.8	1561197.8	1506066.2	48024.0		
	3	1302851.0	1132168.7	1083277.3	1172765.7	115278.9		
**2 hr**	1	1675576.5	2245379.5	2187790.2	2036248.7	313675.7	**1596352.7**	381756.3
	2	1373672.8	1416414.5	1612169.2	1467418.8	127166.1		
	3	1374993.7	1194531.7	1286646.3	1285390.6	90237.6		
**4 hr**	1	2240171.2	2221184.2	2109661.2	2190338.8	70510.9	**1704799.1**	391799.4
	2	1594790.2	1703006.5	1489903.8	1595900.2	106555.7		
	3	1391574.7	1194049.7	1398850.7	1328158.3	116198.5		
**6 hr**	1	2762725.0	2436528.0	2542065.3	2580439.4	166449.8	**1636037.3**	736625.6
	2	1348099.7	1432835.7	1339500.0	1373478.4	51584.4		
	3	911378.7	964951.3	986252.0	954194.0	38578.4		
**8 hr**	1	2167235.3	2054268.3	2607660.7	2276388.1	292397.9	**1700968.4**	468418.5
	2	1613224.0	1500470.3	1452239.3	1521977.9	82619.3		
	3	1364787.0	1227868.3	1320962.3	1304539.2	69921.2		
**16 hr**	1	2585393.3	2684587.7	2725132.7	2665037.9	71891.7	**1699666.3**	727031.5
	2	1221696.3	1236322.3	1344485.0	1267501.2	67069.8		
	3	1154650.3	1171656.0	1173073.3	1166459.9	10251.9		

c. LucF/ LucR ratio in untreated condition

LucF/LucR	Experiment	A	B	C	Mean	SD	Mean	SD	Mean (x100)	SD
	1	0.141	0.132	0.135	0.136	0.004				
	2	0.102	0.099	0.099	0.100	0.001				
**0 hr**	3	0.127	0.115	0.108	0.117	0.010	0.118	0.017	**11.762**	1.653
	1	0.128	0.129	0.123	0.127	0.003				
	2	0.103	0.095	0.091	0.096	0.006				
**30 min**	3	0.111	0.113	0.119	0.114	0.004	0.112	0.014	**11.233**	1.373
	1	0.118	0.122	0.124	0.121	0.003				
	2	0.096	0.090	0.093	0.093	0.003				
**1 hr**	3	0.096	0.102	0.090	0.096	0.006	0.103	0.014	**10.350**	1.397
	1	0.137	0.118	0.122	0.126	0.010				
	2	0.081	0.096	0.088	0.088	0.008				
**2 hr**	3	0.111	0.095	0.088	0.098	0.012	0.104	0.019	**10.406**	1.903
	1	0.131	0.127	0.133	0.130	0.003				
	2	0.089	0.093	0.093	0.092	0.002				
**4 hr**	3	0.117	0.104	0.125	0.115	0.011	0.113	0.018	**11.251**	1.775
	1	0.101	0.123	0.105	0.110	0.012				
	2	0.121	0.106	0.105	0.111	0.009				
**6 hr**	3	0.095	0.058	0.075	0.076	0.018	0.099	0.021	**9.869**	2.089
	1	0.101	0.099	0.104	0.102	0.002				
	2	0.115	0.112	0.123	0.116	0.005				
**8 hr**	3	0.077	0.074	0.084	0.078	0.005	0.099	0.017	**9.879**	1.717
	1	0.101	0.084	0.103	0.096	0.010				
	2	0.110	0.121	0.109	0.113	0.006				
**16 hr**	3	0.082	0.093	0.097	0.091	0.008	0.100	0.012	**10.003**	1.244

d. LucF activity in tunicamycin-treated condition

Firefly	Experiment	A	B	C	Mean	SD	Mean	SD
	1	330238.0	263198.0	315056.3	302830.8	35152.4		
	2	121658.5	107024.5	118294.8	115659.3	7664.7		
**0 hr**	3	125927.2	116417.2	110414.8	117586.4	7822.0	**178692.1**	94910.2
	1	282600.0	322451.0	280620.7	295223.9	23600.1		
	2	113994.8	103369.0	111196.8	109520.2	5507.8		
**30 min**	3	136830.0	166656.5	127822.7	143769.7	20325.8	**182837.9**	87033.1
	1	309715.5	332301.7	313212.3	318409.8	12157.1		
	2	121677.5	148032.8	132898.2	134202.8	13226.0		
**1 hr**	3	121353.5	164486.3	144438.3	143426.1	21584.2	**198679.6**	90976.5
	1	299929.7	260726.7	292908.0	284521.4	20903.8		
	2	159569.5	192234.8	168987.2	173597.2	16813.5		
**2 hr**	3	190302.3	210124.3	216438.0	205621.6	13637.2	**221246.7**	51679.6
	1	347892.0	354078.7	400105.0	367358.6	28527.5		
	2	259245.8	282544.8	238078.2	259956.3	22241.8		
**4 hr**	3	224342.0	291896.7	234274.7	250171.1	36475.1	**292495.3**	61888.8
	1	356302.3	428454.0	302438.3	362398.2	63228.6		
	2	235493.7	232295.3	216127.0	227972.0	10382.0		
**6 hr**	3	127080.0	110351.7	138768.7	125400.1	14282.8	**238590.1**	108038.6
	1	326461.7	261567.7	330131.3	306053.6	38569.6		
	2	244925.3	219002.7	242935.7	235621.2	14426.4		
**8 hr**	3	161368.0	168720.3	187213.0	172433.8	13316.7	**238036.2**	61799.8
	1	171661.7	163881.0	188363.3	174635.3	12509.1		
	2	91344.3	107191.0	99947.3	99494.2	7933.0		
**16 hr**	3	67987.7	70713.3	70440.7	69713.9	1501.2	**114614.5**	47414.3

e. LucR activity in tunicamycin-treated condition

Time	Experiment	A	B	C	Mean	SD	Mean	SD
0 hr	1	2344036.2	1988893.0	2333051.2	2221993.4	201945.6	1461994.1	583372.5
	2	1195947.5	1080045.5	1191256.5	1155749.8	65603.8		
	3	989012.7	1014182.5	1021522.0	1008239.1	17050.1		
30 min	1	2190362.5	2488053.5	2361411.2	2346609.1	149396.5	1611279.6	558289.4
	2	1215983.2	1188291.0	1266115.8	1223463.3	39448.0		
	3	1244480.2	1342541.0	1204277.8	1263766.3	71120.6		
1 hr	1	2519352.5	3296183.2	2840592.2	2885375.9	390346.8	1921587.4	750546.9
	2	1367957.0	1492412.2	1516657.2	1459008.8	79779.5		
	3	1355852.0	1470145.2	1435135.2	1420377.4	58558.3		
2 hr	1	2253047.2	2159190.5	2052057.5	2154765.1	100567.9	1706877.6	355070.3
	2	1536801.2	1712668.5	1368361.8	1539277.2	172166.7		
	3	1490108.5	1420080.2	1369582.8	1426590.5	60526.0		
4 hr	1	1547421.5	1539107.2	1660363.8	1582297.5	67735.1	1415550.4	150149.6
	2	1456116.2	1412693.5	1311993.2	1393600.9	73934.1		
	3	1182851.8	1335946.8	1293459.8	1270752.8	79033.1		
6 hr	1	1872376.7	1964340.0	1684349.3	1840355.3	142715.5	1218155.4	484704.8
	2	1016881.0	1100805.0	960128.7	1025938.2	70774.2		
	3	776874.7	822709.7	764934.0	788172.8	30499.9		
8 hr	1	1351321.3	1165751.7	1358166.7	1291746.6	109168.4	1024160.2	210633.9
	2	910449.0	830987.0	844285.7	861907.2	42561.0		
	3	931342.0	921760.3	903378.3	918826.9	14210.8		
16 hr	1	629519.0	531319.7	574615.0	578484.6	49213.9	402223.1	141018.9
	2	342765.7	385488.7	351502.0	359918.8	22570.9		
	3	277196.7	245075.0	282526.0	268265.9	20259.9		

f. LucF/LucR ratio in tunicamycin-treated condition

Time	Experiment	A	B	C	Mean	SD	Mean	SD	Mean (x100)	SD
**0 hr**	1	0.141	0.132	0.135	0.136	0.004	0.118	0.017	**11.762**	1.653
	2	0.102	0.099	0.099	0.100	0.001				
	3	0.127	0.115	0.108	0.117	0.010				
**30 min**	1	0.129	0.130	0.119	0.126	0.006	0.110	0.017	**10.958**	1.704
	2	0.094	0.087	0.088	0.090	0.004				
	3	0.110	0.124	0.106	0.113	0.009				
**1 hr**	1	0.123	0.101	0.110	0.111	0.011	0.101	0.012	**10.131**	1.196
	2	0.089	0.099	0.088	0.092	0.006				
	3	0.090	0.112	0.101	0.101	0.011				
**2 hr**	1	0.133	0.121	0.143	0.132	0.011	0.130	0.017	**12.999**	1.737
	2	0.104	0.112	0.123	0.113	0.010				
	3	0.128	0.148	0.158	0.145	0.015				
**4 hr**	1	0.225	0.230	0.241	0.232	0.008	0.205	0.024	**20.496**	2.400
	2	0.178	0.200	0.181	0.187	0.012				
	3	0.190	0.218	0.181	0.196	0.020				
**6 hr**	1	0.190	0.218	0.180	0.196	0.020	0.193	0.032	**19.276**	3.187
	2	0.232	0.211	0.225	0.223	0.011				
	3	0.164	0.134	0.181	0.160	0.024				
**8 hr**	1	0.242	0.224	0.243	0.236	0.010	0.233	0.039	**23.254**	3.906
	2	0.269	0.264	0.288	0.273	0.013				
	3	0.173	0.183	0.207	0.188	0.017				
**16 hr**	1	0.273	0.308	0.328	0.303	0.028	0.280	0.026	**28.011**	2.639
	2	0.266	0.278	0.284	0.276	0.009				
	3	0.245	0.289	0.249	0.261	0.024				

g. Endogenous FGF1 expression measured by capillary electrophoresis (4 hr of treatment)

Experiment	UNT	tm	Normalized to UNT
1	176220.0	189739.8	1.08
2	126283.6	121466.3	0.96
3	97719.8	132664.7	1.36
4	170646.9	190332.6	1.12
5	325821.6	356035.9	1.09
6	292197.7	290063.6	0.99
7	143365.8	172500.3	1.20
8	175276.9	210845.4	1.20
9	154490.7	191947.8	1.24
**Mean**	**184669.22**	**206177.38**	**1.14**
SD	55262.41	52980.61	0.10

Table S6. Kinetics of FGF1 IRES activity.

a-f. Cardiomyocytes AC16 transduced with the bicistronic lentivector with the FGF1 IRES were submitted to tunicamycin treatment and the time-course of FGF1 IRES activity (0 hr, 30 min, 1 hr, 2 hr, 4 hr, 6 hr, 8 hr, 16 hr) was evaluated by measuring Firefly/Renilla ratio. Experiments were performed three times with 3 biological replicates (n=9) and each time point was compared to 0 hr time point with a non parametric Mann-Whitney test (Fig. 4b).
g. Endogenous FGF1 expression was measured by capillary electrophoresis, after 4 hr of treatment. 9 independent experiments have been performed.
