# Supplementary material for: Mitochondrial Ribosomal Protein MRPS15 Is a Component of Cytosolic Ribosomes and Regulates Translation in Stressed Cardiomyocytes
Source: Int J Mol Sci. 2024 Mar 13;25(6):3250. doi: 10.3390/ijms25063250 (PMC10970015; doi:10.3390/ijms25063250)
Supplement: Supplementary file 1 [file ijms-25-03250-s001.zip › Supplementary Material/Tables 22 fev 2024/Table S7 Kinetics of IGF1R IRES activity.pdf]

a. **LucF activity in untreated condition**

Time	Experiment	A	B	C	Mean	SD	Mean	SD
	1	601244.2	534885.5	549595.8	561908.5	34850.7		
	2	1025826.8	1044155.5	861535.5	977172.6	100563.1		
**0 hr**	3	1443391.2	1377012.2	1498091.8	1439498.4	60633.6	**992859.8**	316484.1
	1	640894.5	616499.0	713484.8	656959.4	50449.2		
	2	1057663.5	1004997.5	890785.5	984482.2	85309.6		
**30 min**	3	1504549.5	1430436.0	1599150.8	1511378.8	84564.5	**1050940.1**	308453.2
	1	599923.0	705601.5	663877.5	656467.3	53227.5		
	2	1411343.0	1017284.8	1154239.8	1194289.2	200058.6		
**1 hr**	3	1599517.0	1420691.2	1698010.8	1572739.7	140585.6	**1141165.4**	350661.1
	1	729739.5	730748.8	723267.5	727918.6	4059.5		
	2	1113164.2	1266653.2	1403928.5	1261248.6	145457.5		
**2 hr**	3	1354570.5	1644323.2	1627951.8	1542281.8	162768.7	**1177149.7**	313706.4
	1	810873.8	693798.2	771328.5	758666.8	59556.0		
	2	1698900.0	1502423.0	1715199.7	1638840.9	118422.1		
**4 hr**	3	1758892.5	1789613.5	1737642.8	1762049.6	26128.8	**1386519.1**	418568.2
	1	562260.0	499632.7	474672.3	512188.3	45123.5		
	2	1765272.7	1781636.0	1818734.7	1788547.8	27393.0		
**6 hr**	3	1424165.0	1366460.7	1533792.0	1441472.6	84997.7	**1247402.9**	490143.0
	1	481132.0	506849.7	470922.7	486301.4	18513.0		
	2	1848688.0	1937322.3	1868450.7	1884820.3	46529.4		
**8 hr**	3	1360454.7	1398798.0	1557155.0	1438802.6	104273.8	**1269974.8**	522448.9
	1	521239.7	568662.0	566892.0	552264.6	26882.9		
	2	1593295.2	1596525.8	1223091.2	1470970.7	214676.1		
**16 hr**	3	1410000.3	1404281.0	1311218.3	1375166.6	55454.6	**1132800.6**	387024.0

b. LucR activity in untreated condition

Time	Experiment	A	B	C	Mean	SD	Mean	SD
	1	9185735.8	7879593.5	8580291.2	8548540.2	653649.8		
	2	22667079.5	22121995.5	21020088.8	21936387.9	839036.5		
**0 hr**	3	21356100.7	21408528.5	21382924.0	21382517.7	26216.3	**17289148.6**	5827072.3
	1	10294485.8	9744622.0	10607055.8	10215387.9	436623.9		
	2	23429258.8	21272426.5	21584523.5	22095402.9	1165645.7		
**30 min**	3	21496904.0	21035851.0	22799952.0	21777569.0	914927.7	**18029453.3**	5209376.9
	1	9667019.5	10910749.5	10895303.2	10491024.1	713650.7		
	2	26172635.0	24149804.2	25209418.2	25177285.8	1011798.2		
**1 hr**	3	23226380.0	20574263.3	23963343.0	22587995.4	1782446.7	**19418768.4**	5951829.6
	1	11348097.8	11537082.8	11593910.8	11493030.5	128691.4		
	2	24300999.5	25917380.5	24695930.2	24971436.7	842674.2		
**2 hr**	3	18875679.3	23413825.7	23809033.3	22032846.1	2741317.9	**19499104.4**	5475921.5
	1	12510770.5	12051206.5	11864812.2	12142263.1	332466.6		
	2	26812450.3	26232091.3	29181422.3	27408654.7	1562444.6		
**4 hr**	3	24549207.7	26181068.0	24841168.0	25190481.2	870205.1	**21580466.3**	6292135.5
	1	10591288.0	10426924.7	9853200.0	10290470.9	387502.5		
	2	29771388.0	29980437.0	30703271.7	30151698.9	488978.3		
**6 hr**	3	21548339.0	21895102.7	24133966.3	22525802.7	1403461.5	**20989324.1**	7132568.8
	1	9688488.0	10764851.0	9346467.0	9933268.7	740196.9		
	2	29118077.0	31543805.7	31121479.0	30594453.9	1295899.9		
**8 hr**	3	22911251.0	23297957.7	24810383.7	23673197.4	1003633.3	**21400306.7**	7644692.0
	1	10999158.7	10487104.7	10776834.7	10754366.0	256765.4		
	2	25894825.0	27553709.7	24664644.7	26037726.4	1449824.0		
**16 hr**	3	23424593.0	23539252.3	23662685.3	23542176.9	119073.1	**20111423.1**	6238038.1

c. LucF/ LucR ratio in untreated condition

Time	Experiment	A	B	C	Mean	SD	Mean	SD	Mean	SD
	1	0.065	0.068	0.064	0.066	0.002				
	2	0.045	0.047	0.041	0.044	0.003				
**0 hr**	3	0.068	0.064	0.070	0.067	0.003	0.055	0.012	**5.514**	1.191
	1	0.062	0.063	0.067	0.064	0.003				
	2	0.045	0.047	0.041	0.045	0.003				
**30 min**	3	0.070	0.068	0.070	0.069	0.001	0.054	0.011	**5.441**	1.109
	1	0.062	0.065	0.061	0.063	0.002				
	2	0.054	0.042	0.046	0.047	0.006				
**1 hr**	3	0.069	0.069	0.071	0.070	0.001	0.055	0.009	**5.492**	0.928
	1	0.064	0.063	0.062	0.063	0.001				
	2	0.046	0.049	0.057	0.051	0.006				
**2 hr**	3	0.072	0.070	0.068	0.070	0.002	0.057	0.008	**5.693**	0.792
	1	0.065	0.058	0.065	0.062	0.004				
	2	0.063	0.057	0.059	0.060	0.003				
**4 hr**	3	0.072	0.068	0.070	0.070	0.002	0.061	0.004	**6.113**	0.365
	1	0.053	0.048	0.048	0.050	0.003				
	2	0.059	0.059	0.059	0.059	0.000				
**6 hr**	3	0.066	0.062	0.064	0.064	0.002	0.055	0.006	**5.452**	0.557
	1	0.050	0.047	0.050	0.049	0.002				
	2	0.063	0.061	0.060	0.062	0.002				
**8 hr**	3	0.059	0.060	0.063	0.061	0.002	0.055	0.007	**5.535**	0.708
	1	0.047	0.054	0.053	0.051	0.004				
	2	0.062	0.058	0.050	0.056	0.006				
**16 hr**	3	0.060	0.060	0.055	0.058	0.003	0.054	0.005	**5.388**	0.524

d. LucF activity in tunicamycin-treated condition

Time	Experiment	A	B	C	Mean	SD	Mean	SD
	1	601244.2	534885.5	549595.8	561908.5	34850.7		
	2	1025826.8	1044155.5	861535.5	977172.6	100563.1		
**0 hr**	3	1443391.2	1377012.2	1498091.8	1439498.4	60633.6	**992859.8**	316484.1
	1	577832.2	629007.0	585059.2	597299.4	27696.3		
	2	1264769.8	899585.5	1071991.8	1078782.4	182686.8		
**30 min**	3	1405970.8	1458138.5	1576001.8	1480037.1	87105.1	**1052039.6**	337038.8
	1	622262.0	630447.2	612729.5	621812.9	8867.4		
	2	1270410.5	1037199.2	1262080.5	1189896.7	132305.5		
**1 hr**	3	1397949.0	1518652.8	1475992.8	1464198.2	61210.2	**1091969.3**	325608.7
	1	481132.0	506849.7	470922.7	486301.4	18513.0		
	2	1298972.2	1473610.2	1339992.5	1370858.3	91318.8		
**2 hr**	3	1508018.2	1692219.8	1601493.8	1600577.3	92104.3	**1152579.0**	444185.0
	1	790249.8	602737.5	595327.8	662771.7	110461.4		
	2	1728486.0	1659576.0	1681873.7	1689978.6	35162.7		
**4 hr**	3	1800019.5	1799203.2	1737139.8	1778787.5	36070.2	**1377179.3**	476271.7
	1	498812.3	441356.3	490051.0	476739.9	30954.6		
	2	1700196.3	1860079.3	2047234.7	1869170.1	173697.7		
**6 hr**	3	1457490.7	1421321.7	1513486.0	1464099.4	46436.2	**1270003.1**	528842.2
	1	425337.7	393006.7	373172.3	397172.2	26331.0		
	2	1707848.0	1912179.0	1805489.0	1808505.3	102198.9		
**8 hr**	3	1174501.3	1483493.3	1458668.3	1372221.0	171679.6	**1192632.9**	534336.3
	1	522393.2	484535.5	442786.5	483238.4	39819.2		
	2	918053.8	830863.8	923647.5	890855.1	52029.1		
**16 hr**	3	658711.7	712375.0	726085.7	699057.4	35606.6	**691050.3**	145727.6

e. LucR activity in tunicamycin-treated condition

Time	Experiment	A	B	C	Mean	SD	Mean	SD
0 hr	1	9185735.8	7879593.5	8580291.2	8548540.2	653649.8		
	2	22667079.5	22121995.5	21020088.8	21936387.9	839036.5		
	3	21356100.7	21408528.5	21382924.0	21382517.7	26216.3	**17289148.6**	5827072.3
30 min	1	9922533.8	10229378.5	10424279.5	10192063.9	252945.6		
	2	25970134.8	22263989.5	24831936.2	24355353.5	1898480.1		
	3	21211819.7	21432298.5	23563466.3	22069194.8	1298764.1	**18872204.1**	5786760.1
1 hr	1	10819362.5	11192227.2	11186717.2	11066102.3	213700.7		
	2	25291560.5	25297793.8	25526334.5	25371896.3	133783.7		
	3	22045784.5	22469365.0	22800934.3	22438694.6	378508.0	**19625564.4**	5706308.1
2 hr	1	10672629.3	9425847.3	8631733.3	9576736.7	1028780.7		
	2	25404842.2	25943573.8	24164444.5	25170953.5	912333.9		
	3	18946629.0	22767159.3	21416462.7	21043417.0	1937391.4	**18597035.7**	6013532.7
4 hr	1	9157618.2	8874163.2	8352043.5	8794608.3	408637.2		
	2	22566264.0	21722719.3	22465246.3	22251409.9	460637.0		
	3	19302674.7	19236560.3	17437016.0	18658750.3	1058569.3	**16568256.2**	5182431.9
6 hr	1	5707557.7	6468723.3	6793851.3	6323377.4	557541.5		
	2	18797739.3	19533741.3	22025645.7	20119042.1	1691678.9		
	3	14580117.7	14335709.3	14548020.3	14487949.1	132816.7	**13643456.2**	4880052.5
8 hr	1	4929376.3	4938396.0	4512184.3	4793318.9	243511.4		
	2	15530515.3	18023923.0	17064235.7	16872891.3	1257668.5		
	3	10512410.0	12480250.0	12415403.0	11802687.7	1117883.5	**11156299.3**	4385073.4
16 hr	1	3249819.8	3223857.8	2744256.2	3072644.6	284688.8		
	2	5896815.2	5583069.5	5932838.5	5804241.1	192385.2		
	3	4691651.3	4763560.7	4971224.7	4808812.2	145176.1	**4561899.3**	992836.5

f. LucF/ LucR ratio in tunicamycin-treated condition

Time	Experiment	A	B	C	Mean	SD	Mean	SD	Mean	SD
	1	0.065	0.068	0.064	0.066	0.002				
	2	0.045	0.047	0.041	0.044	0.003				
**0 hr**	3	0.068	0.064	0.070	0.067	0.003	0.055	0.012	**5.514**	1.191
	1	0.058	0.061	0.056	0.059	0.003				
	2	0.049	0.040	0.043	0.044	0.004				
**30 min**	3	0.066	0.068	0.067	0.067	0.001	0.051	0.009	**5.135**	0.856
	1	0.058	0.056	0.055	0.056	0.001				
	2	0.050	0.041	0.049	0.047	0.005				
**1 hr**	3	0.063	0.068	0.065	0.065	0.002	0.052	0.006	**5.155**	0.610
	1	0.045	0.054	0.055	0.051	0.005				
	2	0.051	0.057	0.055	0.054	0.003				
**2 hr**	3	0.080	0.074	0.075	0.076	0.003	0.053	0.004	**5.280**	0.423
	1	0.086	0.068	0.071	0.075	0.010				
	2	0.077	0.076	0.075	0.076	0.001				
**4 hr**	3	0.093	0.094	0.100	0.095	0.004	0.076	0.006	**7.556**	0.623
	1	0.087	0.068	0.072	0.076	0.010				
	2	0.090	0.095	0.093	0.093	0.002				
**6 hr**	3	0.100	0.099	0.104	0.101	0.003	0.084	0.011	**8.440**	1.138
	1	0.086	0.080	0.083	0.083	0.003				
	2	0.110	0.106	0.106	0.107	0.002				
**8 hr**	3	0.112	0.119	0.117	0.116	0.004	0.095	0.014	**9.507**	1.363
**16 h**	1	0.161	0.150	0.161	0.157	0.006	0.155	0.005	**15.543**	0.517

Table S7. Kinetics of IGF1R IRES activity

Cardiomyocytes AC16 transduced with the bicistronic lentivector with the IGF1R IRES were submitted to tunicamycin treatment and the time-course of IGF1R IRES activity (0 hr, 30 min, 1 hr, 2 hr, 4 hr, 6 hr, 8 hr, 16 hr) was evaluated by measuring Firefly/Renilla ratio. Experiments were performed three times with 3 biological replicates (n=9) and each time point was compared to 0 hr time point with a non parametric Mann-Whitney test (Fig. S1).
