# Supplementary material for: Mitochondrial Ribosomal Protein MRPS15 Is a Component of Cytosolic Ribosomes and Regulates Translation in Stressed Cardiomyocytes
Source: Int J Mol Sci. 2024 Mar 13;25(6):3250. doi: 10.3390/ijms25063250 (PMC10970015; doi:10.3390/ijms25063250)
Supplement: Supplementary file 1 [file ijms-25-03250-s001.zip › Supplementary Material/Tables 22 fev 2024/Table S8 MRPS15 knock-down.pdf]

a. MRPS15 expression (AU)

Condition	Sample	Experiment						Mean	SD
		1	2	3	4	5	6		
**unt**	Si-	68294.6	87855.3	71193.2	28117.0	42069.0	137620.8	**72525.0**	26808.7
	Si MRPS15	51161.7	50953.2	76298.7	20481.3	32556.3	120528.9	**58663.4**	26500.3
**tm**	Si-		29609.1	91584.3	48488.5	52193.0	85240.0	**61423.0**	21591.3
	Si MRPS15		21309.5	85577.6	69216.5	35295.6	48560.4	**51991.9**	20324.1

b. MRPS15 expression normalized to Si-

Condition	Sample	Experiment						Mean	SD
		1	2	3	4	5	6		
**unt**	Si-	0.94	1.21	0.98	0.39	0.58	1.90	**1.00**	0.37
	Si MRPS15	0.71	0.70	1.05	0.28	0.45	1.66	**0.81**	0.37
**tm**	Si-		0.48	1.49	0.79	0.85	1.39	**1.00**	0.35
	Si MRPS15		0.35	1.39	1.13	0.57	0.79	**0.85**	0.33

c. Bicistronic mRNA with FGF1 IRES : LucF activity

Condition	Sample	Experiment	A	B	C	Mean	SD
		1	242297.3	233101.0	241521.7		
		2	322063.3	312528.3	314891.3		
		3	182708.0	221168.3	223989.3		
	Si-	4	255196.3	303162.3	295521.3	**262345.7**	39406.3
**unt**		1	124823.3	120786.7	95192.0		
		2	182340.7	166837.0	168049.3		
		3	160841.0	234414.0	256492.3		
	Si MRPS15	4	218627.0	321815.3	306891.7	**196425.9**	59351.8
		1	363242.0	326820.7	285572.0		
		2	406631.7	363396.0	348966.7		
		3	313251.0	360163.0	325421.7		
	Si-	4	352380.0	379283.3	367418.3	**349378.9**	24477.1
**tm**		1	138137.0	140459.0	163327.3		
		2	223652.7	193383.3	201106.7		
		3	277270.7	304662.0	265350.3		
	Si MRPS15	4	415622.7	386190.3	273580.3	**248561.9**	71884.2

d. Bicistronic mRNA with FGF1 IRES : LucR activity

Condition	Sample	Experiment	A	B	C	Moyenne	SD
		1	242297.3	233101.0	241521.7		
		2	3454086.3	3171775.7	3374068.0		
		3	1789209.7	2256620.3	2439872.3		
	Si-	4	2185324.0	3016508.3	2545331.3	**2079143.0**	968407.1
**unt**		1	124823.3	120786.7	95192.0		
		2	2172762.0	2052839.0	2205825.3		
		3	1614655.7	2321493.7	2565375.0		
	Si MRPS15	4	1964949.7	2975219.0	3148760.0	**1780223.4**	860906.0
		1	363242.0	326820.7	285572.0		
		2	2573645.3	2341658.0	2161048.7		
		3	1704756.3	1946629.0	1693801.7		
	Si-	4	2100751.7	2111874.0	1935621.7	**1628785.1**	651786.8
**tm**		1	138137.0	140459.0	163327.3		
		2	1682956.3	1371043.0	1389566.7		
		3	1792124.3	1893398.7	1553409.0		
	Si MRPS15	4	2777367.7	2124170.0	1577144.7	**1383592.0**	620233.6

e. Bicistronic mRNA with FGF1 IRES : LucF/ LucR ratio

Condition	Sample	Experiment	A	B	C	Mean	SD	Mean x100	SD
unt	Si-	1	0.097	0.109	0.090				
		2	0.093	0.099	0.093				
		3	0.102	0.098	0.092				
		4	0.117	0.101	0.116	0.100	0.007	**10.047**	0.695
	Si MRPS15	1	0.096	0.091	0.079				
		2	0.084	0.081	0.076				
		3	0.100	0.101	0.100				
		4	0.111	0.108	0.097	0.094	0.010	**9.378**	0.955
tm	Si-	1	0.190	0.198	0.188				
		2	0.158	0.155	0.161				
		3	0.184	0.185	0.192				
		4	0.168	0.180	0.190	0.179	0.012	**17.910**	1.233
	Si MRPS15	1	0.167	0.170	0.164				
		2	0.133	0.141	0.145				
		3	0.155	0.161	0.171				
		4	0.150	0.182	0.173	0.159	0.012	**15.930**	1.225

f. Bicistronic mRNA with a hairpin : LucF activity

Condition	Sample	Experiment	A	B	C	Mean	SD
unt	Si-	1	317308.3	315326.7	315837.7	**232368.5**	55859.4
		2	193504.3	196571.0	199433.0		
		3	163875.0	186669.3	202791.0		
	Si MRPS15	1	124354.0	157638.7	155482.7	**182301.6**	29236.9
		2	175063.0	195892.0	199808.0		
		3	167403.3	251739.7	213332.7		
tm	Si-	1	402318.0	361439.0	317126.0	**248447.0**	74564.9
		2	201913.0	173321.3	174252.7		
		3	213780.0	215829.3	176044.0		
	Si MRPS15	1	175463.0	118725.3	126208.3	**186128.1**	33569.2
		2	203615.7	224643.7	173054.7		
		3	234116.7	208701.0	210625.0		

g. Bicistronic mRNA with a hairpin : LucR activity

Condition	Sample	Experiment	A	B	C	Mean	SD
unt	Si-	1	8835174.7	8546045.3	7955712.3	5794113.3	1767687.2
		2	4042809.0	4359879.3	4463674.7		
		3	4875088.0	4734438.3	4334198.3		
	Si MRPS15	1	3747066.0	4529719.3	4396046.0	4798339.6	776255.9
		2	4327529.7	4099460.3	4197065.0		
		3	6181961.7	5786070.7	5920138.0		
tm	Si-	1	6529759.0	5729023.3	5865106.7	4344818.7	1130985.1
		2	3651268.7	3172034.3	3431234.7		
		3	3657035.3	3474025.3	3593880.7		
	Si MRPS15	1	3094594.0	2350165.3	2295466.7	3280997.6	525819.9
		2	3478618.0	3380043.7	3017575.0		
		3	4486510.3	4071462.0	3354543.3		

h. Bicistronic mRNA with a hairpin : LucF/ LucR ratio

Condition	Sample	Experiment	A	B	C	Mean	SD	Mean (x100)	SD
**unt**	Si-	1	0.036	0.037	0.040	0.041	0.004	**0.041**	0.004
		2	0.048	0.045	0.045				
		3	0.034	0.039	0.047				
	Si MRPS15	1	0.033	0.035	0.035	0.038	0.006	**0.038**	0.006
		2	0.040	0.048	0.048				
		3	0.027	0.044	0.036				
**tm**	Si-	1	0.062	0.063	0.054	0.057	0.004	**0.057**	0.004
		2	0.055	0.055	0.051				
		3	0.058	0.062	0.049				
	Si MRPS15	1	0.057	0.051	0.055	0.057	0.004	**0.057**	0.004
		2	0.059	0.066	0.057				
		3	0.052	0.051	0.063				

i. Endogenous FGF1 expression (AU)

Condition	Sample	Experiment						Mean	SD
		1	2	3	4		5		
unt	Si-	176875.2	134600.7	116210.8	141413.3		474705.0	**208761.0**	106377.6
	Si MRPS15	165304.5	120992.9	118454.7	151121.9	159686.1	404502.0	**186677.0**	72608.3
tm	Si-	168838.1	114327.3	211466.0	165122.4		438622.4	**219675.2**	87578.9
	Si MRPS15	170666.7	107918.0	143150.8	120814.8	144252.9	427373.0	**185696.0**	80559.0

j. Endogenous FGF1 expression normalized to Si-

Condition	Sample	Experiment						Mean	SD
		1	2	3	4		5		
unt	Si-	0.85	0.64	0.56	0.68		2.27	**1.00**	0.51
	Si MRPS15	0.79	0.58	0.57	0.72	0.76	1.94	**0.89**	0.35
tm	Si-	0.77	0.52	0.96	0.75		2.00	**1.00**	0.40
	Si MRPS15	0.78	0.49	0.65	0.55	0.66	1.95	**0.85**	0.37

Table S8. Effect of MRPS15 knock-down on IRES activity.

a-b) Cells were transfected with MRPS15 siRNA and protein depletion was measured after 48 hr by capillary electrophoresis Simple Western (Fig. 5a).
c-h) FGF1 IRES activity and hairpin control activity were obtained by measuring Firefly/Renilla ratio, the experiment was performed 4 times with 3 biological replicates (n=12) for the FGF1 IRES and 3 times (n=9) for the hairpin (Fig. 5b). Data were pooled and showed in a histogram corresponding to the mean +/- SEM. Data of SiMRPS15 transfection were compared to corresponding Si control with a 2way ANOVA test.
i-j) Endogeneous FGF1 expression was analyzed by capillary electrophoresis. 6 independent experiments were performed (Fig. 5c).
