# Supplementary material for: Mitochondrial Ribosomal Protein MRPS15 Is a Component of Cytosolic Ribosomes and Regulates Translation in Stressed Cardiomyocytes
Source: Int J Mol Sci. 2024 Mar 13;25(6):3250. doi: 10.3390/ijms25063250 (PMC10970015; doi:10.3390/ijms25063250)
Supplement: Supplementary file 1 [file ijms-25-03250-s001.zip › Supplementary Material/Tables 22 fev 2024/Table S9 MRPS15 overexpression.pdf]

a. MRPS15 overexpression (AU)

Condition	Sample	Experiment								Mean	SD
		1	2	3	4	5	6	7	8		
**unt**	Ctrl	74608.1	115702.5	76231.2	1090.5	10378.7	72716.3			**58454.6**	35146.6
	+ cMRPS15	99683.3	118213.8	33387.7	17860.3	22156.7	120864.8	86416.3	88205.9	**73348.6**	36660.3
**tm**	Ctrl	103786.3	98110.3	44773.4	6624.3	7985.4	77991.4			**56545.2**	36750.8
	+ cMRPS15	135055.3	61547.1	69374.6	5123.6	7855.6	77592.9	103561.6	98329.3	**69805.0**	33829.8

b. MRPS15 overexpression normalized to NT

Condition	Sample	Experiment								Mean	SD
		1	2	3	4	5	6	7	8		
**unt**	Ctrl	1.28	1.98	1.30	0.02	0.18	1.24			**1.00**	0.60
	+ cMRPS15	1.71	2.02	0.57	0.31	0.38	2.07	1.48	1.51	**1.25**	0.63
**tm**	Ctrl	1.84	1.74	0.79	0.12	0.14	1.38			**1.00**	0.65
	+ cMRPS15	2.39	1.09	1.23	0.09	0.14	1.37	1.83	1.74	**1.23**	0.60

c. Bicistronic mRNA with FGF1 IRES : LucF activity

Condition	Sample	Experiment	A	B	C	Mean	SD
**unt**	Ctrl	1	390930.0	334163.3	334988.3	**108156.5**	122602.0
		2	22939.7	26629.3	21690.0		
		3	25945.3	26522.0	26280.3		
		4	24720.7	24879.7	38189.2		
	+ cMRPS15	1	373113.0	268811.7	271078.3	**67571.2**	78921.0
		2	29090.3	25298.7	24734.3		
			19259.7	12885.7	17999.7		
		3	24783.7	22600.3	24395.0		
			15498.7	15741.7	19216.0		
		4	16416.7	18871.7	16487.0		
**tm**	Ctrl	1	330645.0	448461.0	379019.3	**123033.7**	131504.0
		2	34656.7	36246.0	28409.3		
		3	39083.3	34783.7	31702.3		
		4	38220.7	39609.3	35567.7		
	+ cMRPS15	1	325793.0	354985.7	264589.7	**76714.9**	79469.3
		2	38626.7	35073.3	31535.7		
			27183.0	20626.0	20220.7		
		3	40598.0	34077.7	37689.0		
			23244.7	24074.7	21205.0		
		4	25598.0	27521.3	28225.3		

d. Bicistronic mRNA with FGF1 IRES : LucR activity

Condition	Sample	Experiment	A	B	C	Mean	SD
**unt**	Ctrl	1	5382741.0	5570912.7	5503074.3	**1660248.4**	1912663.8
		2	344686.3	373148.0	357865.7		
		3	310763.0	377055.0	388159.0		
		4	340433.7	403480.7	570661.8		
	+ cMRPS15	1	4652681.7	4064922.0	3447235.7	**914482.0**	1046821.5
		2	374864.7	359798.0	352597.0		
			251211.3	213511.7	278753.3		
		3	341894.7	354414.7	387360.7		
			200175.7	231530.7	222982.0		
		4	224080.7	232906.3	269754.7		
**tm**	Ctrl	1	4770904.0	4775582.3	4719086.7	**1406328.7**	1674431.2
		2	304204.0	296786.3	249560.7		
		3	287103.0	257949.7	240724.7		
		4	331517.3	345958.7	296566.7		
	+ cMRPS15	1	4099537.7	3512178.7	3166935.3	**782072.7**	936937.1
		2	318958.0	283693.7	271228.7		
			213010.0	167560.0	166890.3		
		3	279656.7	243728.0	262560.0		
			165937.0	162368.3	145298.3		
		4	213762.0	213539.0	190466.3		

e. Bicistronic mRNA with FGF1 IRES : LucF/ LucR ratio

Condition	Sample	Experiment	A	B	C	Mean	SD	Mean (x100)	SD
**unt**	Ctrl	1	0.0726	0.0600	0.0609	0.0679	0.0052	**6.7895**	0.5160
		2	0.0666	0.0714	0.0606				
		3	0.0835	0.0703	0.0677				
		4	0.0726	0.0617	0.0669				
	+ cMRPS15	1	0.0802	0.0661	0.0786	0.0717	0.0065	**7.1714**	0.6451
		2	0.0776	0.0703	0.0701				
			0.0767	0.0604	0.0646				
		3	0.0725	0.0638	0.0630				
			0.0774	0.0680	0.0862				
		4	0.0733	0.0810	0.0611				
**tm**	Ctrl	1	0.0693	0.0939	0.0803	0.1122	0.0155	**11.2150**	1.5487
		2	0.1139	0.1221	0.1138				
		3	0.1361	0.1348	0.1317				
		4	0.1153	0.1145	0.1199				
	+ cMRPS15	1	0.0795	0.1011	0.0835	0.1254	0.0155	**12.5368**	1.5467
		2	0.1211	0.1236	0.1163				
			0.1276	0.1231	0.1212				
		3	0.1452	0.1398	0.1435				
			0.1401	0.1483	0.1459				
		4	0.1198	0.1289	0.1482				

f. Bicistronic mRNA with IGF1R IRES : LucF activity

Condition	Sample	Experiment	A	B	C	Mean	SD
unt	Ctrl	1	128481.3	122097.7	118701.7	**112076.2**	16388.7
		2	74221.7	86889.0	101369.0		
		3	119512.0	120280.0	137133.7		
	+ cMRPS15	1	147833.3	141550.0	110944.3	**130652.6**	18877.9
		2	122181.7	104296.3	100237.3		
		3	159110.0	148559.0	141161.0		
tm	Ctrl	1	103537.0	105539.7	94827.0	**105184.4**	13025.2
		2	88138.5	85813.2	94993.2		
		3	123570.3	126618.3	123622.7		
	+ cMRPS15	1	150042.7	117780.7	142700.0	**132973.2**	14754.7
		2	105806.5	120851.8	128907.5		
		3	156729.7	125123.7	148816.7		

g. Bicistronic mRNA with IGF1R IRES : LucR activity

Condition	Sample	Experiment	A	B	C	Mean	SD
unt	Ctrl	1	2509851.3	2513133.7	2452448.0	**2593799.6**	492432.2
		2	1757701.3	1978385.0	2135333.0		
		3	3156681.7	3267478.0	3573184.0		
	+ cMRPS15	1	2815105.3	2750954.0	2437360.3	**2754442.2**	391381.6
		2	2340292.7	2151969.7	2330417.3		
		3	3379139.0	3300914.0	3283827.7		
tm	Ctrl	1	1739531.7	1731717.0	1431858.3	**1841381.4**	408580.1
		2	1451151.5	1450547.2	1404871.8		
		3	2451635.0	2469095.0	2442024.7		
	+ cMRPS15	1	2141836.7	1733093.0	1944261.7	**2029041.5**	312441.1
		2	1561815.8	1757877.8	1742174.2		
		3	2654214.3	2203373.7	2522726.3		

h. Bicistronic mRNA with IGF1R IRES : LucF/ LucR ratio

Condition	Sample	Experiment	A	B	C	Mean (x100)	SD
unt	Ctrl	1	0.0512	0.0486	0.0484	**0.0439**	0.0045
		2	0.0422	0.0439	0.0475		
		3	0.0379	0.0368	0.0384		
	+ cMRPS15	1	0.0525	0.0515	0.0455	**0.0476**	0.0032
		2	0.0522	0.0485	0.0430		
		3	0.0471	0.0450	0.0430		
tm	Ctrl	1	0.0595	0.0609	0.0662	**0.0585**	0.0052
		2	0.0607	0.0592	0.0676		
		3	0.0504	0.0513	0.0506		
	+ cMRPS15	1	0.0701	0.0680	0.0734	**0.0663**	0.0054
		2	0.0677	0.0687	0.0740		
		3	0.0590	0.0568	0.0590		

i. Bicistronic mRNA with VEGFD IRES : LucF activity

Condition	Sample	Experiment	A	B	C	Mean	SD
unt	Ctrl	1	82814.3	84593.3	93684.3	**61589.2**	13436.6
		2	59241.3	61179.3	65884.3		
		3	47734.7	42754.7	50566.7		
		4	46025.0	51416.0	53176.7		
	+ cMRPS15	1	72956.0	64602.0	67981.0	**47919.8**	12877.8
		2	54432.3	53884.7	50929.3		
		3	38130.3	34895.7	35855.7		
		4	30489.0	35349.7	35531.7		
tm	Ctrl	1	134000.7	142765.3	109008.7	**90646.3**	20720.9
		2	96791.3	94991.3	82296.7		
		3	75487.5	75646.5	65142.8		
		4	76945.2	68590.5	66089.5		
	+ cMRPS15	1	129937.3	112369.0	114124.0	**80849.1**	22725.2
		2	99947.0	84219.3	70758.0		
		3	59843.2	60071.8	52976.5		
		4	64954.2	63827.2	57161.5		

j. Bicistronic mRNA with VEGFD IRES : LucR activity

Condition	Sample	Experiment	A	B	C	Mean	SD
unt	Ctrl	1	2915394.7	3098664.0	3396357.7	**2324410.8**	460543.3
		2	2420258.0	2202914.7	2554639.7		
		3	2007703.0	2015707.3	2051070.7		
		4	1680429.7	1721852.0	1827938.7		
	+ cMRPS15	1	2685596.7	2491582.3	2719482.7	**1912992.6**	460975.1
		2	2212691.3	2044759.0	2089694.3		
		3	1639983.7	1524923.0	1480133.3		
		4	1217955.7	1393440.7	1455668.3		
tm	Ctrl	1	1858093.7	1855202.3	1466665.7	**1362882.6**	237846.6
		2	1591667.7	1457804.3	1374941.3		
		3	1247386.3	1225042.7	1109500.0		
		4	1121795.7	1063435.7	983055.7		
	+ cMRPS15	1	1637169.7	1405105.3	1392799.3	**1147486.9**	238753.0
		2	1471253.7	1263624.7	1112736.3		
		3	999273.7	919031.3	875278.3		
		4	921368.7	932948.3	839253.3		

k. Bicistronic mRNA with VEGFD IRES : LucF/LucR ratio

Condition	Sample	Experiment	A	B	C	Mean	SD	Mean (x100)	SD
**unt**	Ctrl	1	0.028	0.027	0.028	0.026	0.002	**2.644**	0.205
		2	0.024	0.028	0.026				
		3	0.024	0.021	0.025				
		4	0.027	0.030	0.029				
	+ cMRPS15	1	0.027	0.026	0.025	0.025	0.001	**2.488**	0.093
		2	0.025	0.026	0.024				
		3	0.023	0.023	0.024				
		4	0.025	0.025	0.024				
**tm**	Ctrl	1	0.072	0.077	0.074	0.066	0.005	**6.588**	0.497
		2	0.061	0.065	0.060				
		3	0.061	0.062	0.059				
		4	0.069	0.064	0.067				
	+ cMRPS15	1	0.079	0.080	0.082	0.069	0.006	**6.935**	0.573
		2	0.068	0.067	0.064				
		3	0.060	0.065	0.061				
		4	0.070	0.068	0.068				

l. Bicistronic mRNA with a hairpin : LucF activity

Condition	Sample	Experiment	A	B	C	Mean	SD
unt	Ctrl	1	271416.3	211328.0	247233.7	**121922.9**	122772.7
		2	17849.7	12818.7	15160.7		
		3	9444.3	16654.0	11530.0		
	+ cMRPS15	1	197213.3	346335.3	261855.7	**99545.4**	112615.1
		2	21565.3	13481.3	16566.3		
		3	12204.7	12340.0	14346.7		
tm	Ctrl	1	324817.0	280777.7	245442.0	**107288.3**	117593.7
		2	21977.3	21549.0	16820.0		
		3	13594.8	21078.2	19538.8		
	+ cMRPS15	1	452029.3	226456.7	192854.3	**108354.6**	121394.8
		2	17462.7	23387.0	17502.3		
		3	13307.5	15917.2	16274.8		

m. Bicistronic mRNA with a hairpin : LucR activity

Condition	Sample	Experiment	A	B	C	Mean	SD
unt	Ctrl	1	9983504.0	9762276.7	9390271.3	**3214216.5**	3504813.3
		2	695288.3	714624.0	839212.7		
		3	495188.7	629031.0	609596.0		
	+ cMRPS15	1	7698981.0	7454499.7	5930942.0	**2780750.7**	2831593.4
		2	726072.3	686454.3	759822.3		
		3	575450.3	582235.3	612299.3		
tm	Ctrl	1	8452577.3	9324106.3	8245770.0	**3182010.6**	3661427.1
		2	564971.0	499836.3	456918.7		
		3	325398.2	346725.2	421792.2		
	+ cMRPS15	1	7541620.7	6832099.0	5495442.0	**2505523.6**	2376605.5
		2	494086.7	532249.7	472121.7		
		3	383290.5	380769.8	418032.5		

n. Bicistronic mRNA with a hairpin : LucF/ LucR ratio

Condition	Sample	Experiment	A	B	C	Mean	SD	Mean (x100)	SD
**unt**	Ctrl	1	0.027	0.022	0.026	0.030	0.009	**2.237**	0.360
		2	0.026	0.018	0.018				
		3	0.019	0.026	0.019				
	+ cMRPS15	1	0.026	0.046	0.044	0.028	0.008	**2.813**	0.798
		2	0.030	0.020	0.022				
		3	0.021	0.021	0.023				
**tm**	Ctrl	1	0.038	0.030	0.030	0.041	0.007	**4.067**	0.652
		2	0.039	0.043	0.037				
		3	0.042	0.061	0.046				
	+ cMRPS15	1	0.060	0.033	0.035	0.040	0.006	**4.000**	0.571
		2	0.035	0.044	0.037				
		3	0.035	0.042	0.039				

o. Bicistronic mRNA with EMCV IRES : LucF activity

Condition	Sample	Experiment	A	B	C	Mean	SD
unt	Ctrl	1	276116.0	300373.0	270385.3	197070.9	54073.5
		2	226086.0	218083.3	215822.3		
		3	143090.3	141096.7	135366.3		
		4	143472.0	150001.3	144957.7		
	+ cMRPS15	1	234156.0	243117.7	206244.3	177305.1	33061.7
		2	206987.7	183428.3	188266.7		
		3	142691.0	138443.0	147237.0		
		4	132148.7	158426.7	146514.0		
tm	Ctrl	1	204604.3	210760.7	160983.7	130030.5	38372.5
		2	174008.7	124069.3	127585.3		
		3	91941.8	95721.5	89518.2		
		4	98377.5	87125.2	95670.2		
	+ cMRPS15	1	202261.0	171990.3	154257.0	129126.5	26263.4
		2	140945.7	133758.7	122003.7		
		3	99928.5	97587.8	84668.5		
		4	118723.5	123318.2	100075.2		

p. Bicistronic mRNA with EMCV IRES : LucR activity

Condition	Sample	Experiment	A	B	C	Mean	SD
**unt**	Ctrl	1	737771.7	826047.7	778817.3	**529513.0**	153967.3
		2	531313.0	603988.0	622944.0		
		3	385115.3	400423.0	379762.7		
		4	346159.0	366412.3	375402.0		
	+ cMRPS15	1	615802.0	680740.0	569639.0	**472256.4**	88942.1
		2	502485.0	477541.7	520983.3		
		3	376392.3	409643.3	386047.0		
		4	357433.7	396426.0	373943.3		
**tm**	Ctrl	1	465142.0	479308.3	372283.7	**297745.3**	94178.1
		2	408792.3	306497.3	319516.7		
		3	212853.0	224984.7	210641.0		
		4	200143.8	181143.5	191637.2		
	+ cMRPS15	1	419367.0	389527.0	305729.0	**284161.7**	61356.5
		2	342666.0	331658.7	272334.3		
		3	223391.7	230672.0	198728.7		
		4	234483.5	252780.5	208602.2		

q. Bicistronic mRNA with EMCV IRES : LucF/ LucR ratio

Condition	Sample	Experiment	A	B	C	Mean	SD	Mean	SD
unt	Ctrl	1	0.374	0.364	0.347				
		2	0.426	0.361	0.346				
		3	0.372	0.352	0.356				
		4	0.414	0.409	0.386	0.376	0.022	**37.571**	2.211
	+ cMRPS15	1	0.380	0.357	0.362				
		2	0.412	0.384	0.361				
		3	0.379	0.338	0.381				
		4	0.370	0.400	0.392	0.376	0.016	**37.637**	1.560
tm	Ctrl	1	0.440	0.440	0.432				
		2	0.426	0.405	0.399				
		3	0.432	0.425	0.425				
		4	0.492	0.481	0.499	0.441	0.025	**44.133**	2.463
	+ cMRPS15	1	0.482	0.442	0.505	0.455	0.031	**45.511**	3.087

Table S9. Effect of MRPS15 overexpression on IRES activities.

a-b) AC16 cells were transduced with the lentivector overexpressing cMRPS15 and with bicistronic IRES-containing lentivectors (FGF1, IGF1R, VEGFD, EMCV IRESs and hairpin control). MRPS15 expression was measured by capillary electrophoresis, a representative experiment and a histogram showing the mean +/- SEM of 3 independent experiments is shown (Fig. 6b). c-q) IRESs activity was obtained by measuring Firefly/Renilla ratio, for each IRES experiments were performed at least 3 times with 3 biological replicates (n≥9) and IRES activity of MRPS15 overexpression was compared to WT in corresponding conditions (untreated or treated tunicamycin) using a 2way ANOVA test (Fig. 6c).
