# Supplementary figures and images for: Mitochondrial Ribosomal Protein MRPS15 Is a Component of Cytosolic Ribosomes and Regulates Translation in Stressed Cardiomyocytes
Source: Int J Mol Sci. 2024 Mar 13;25(6):3250. doi: 10.3390/ijms25063250 (PMC10970015; doi:10.3390/ijms25063250)

## Slide 1
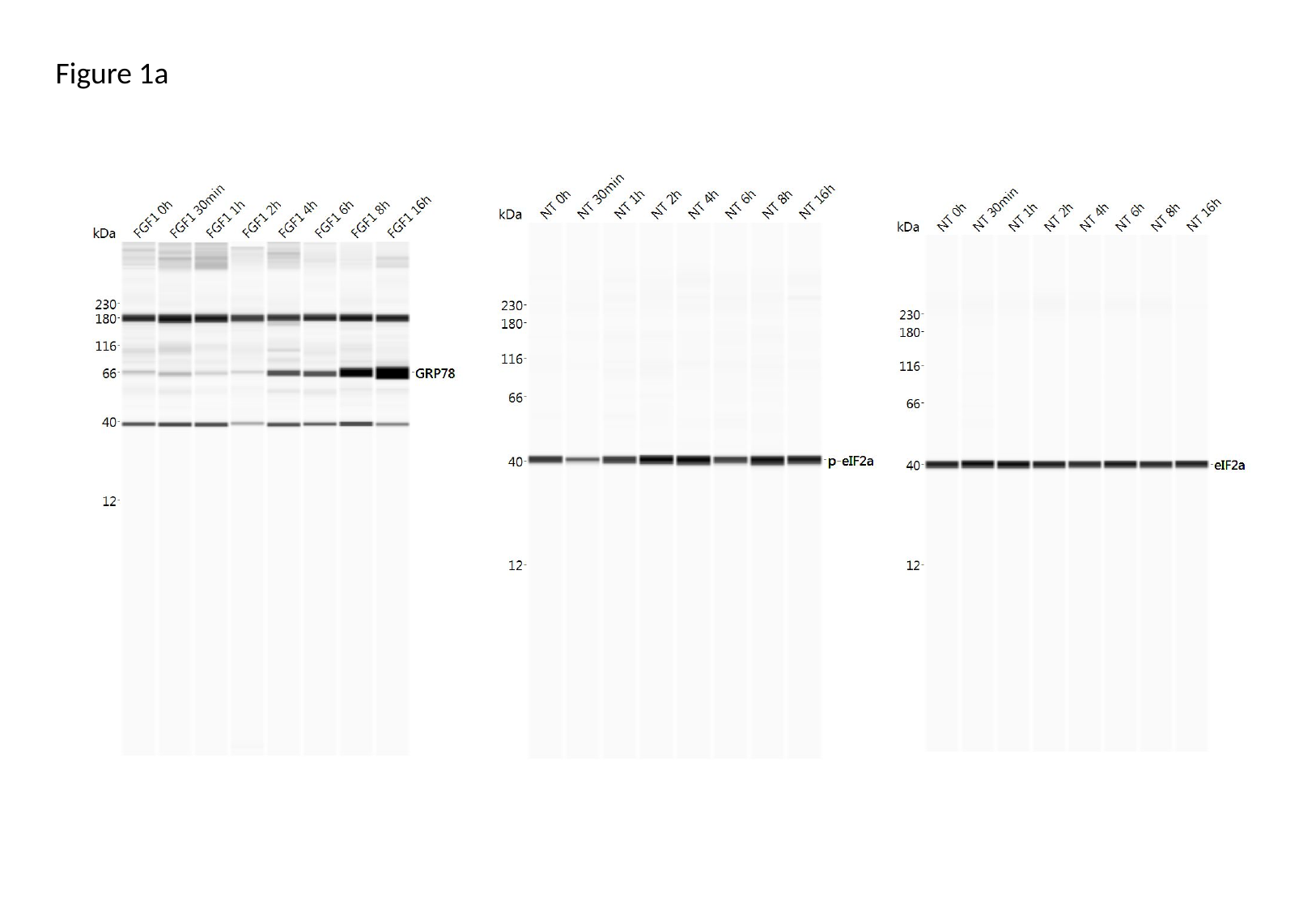

Figure 1a

Supplement: Supplementary file 1 [file ijms-25-03250-s001.zip › Supplementary Material/Figure 1 Full Blots (Jess).pptx]

## Slide 1
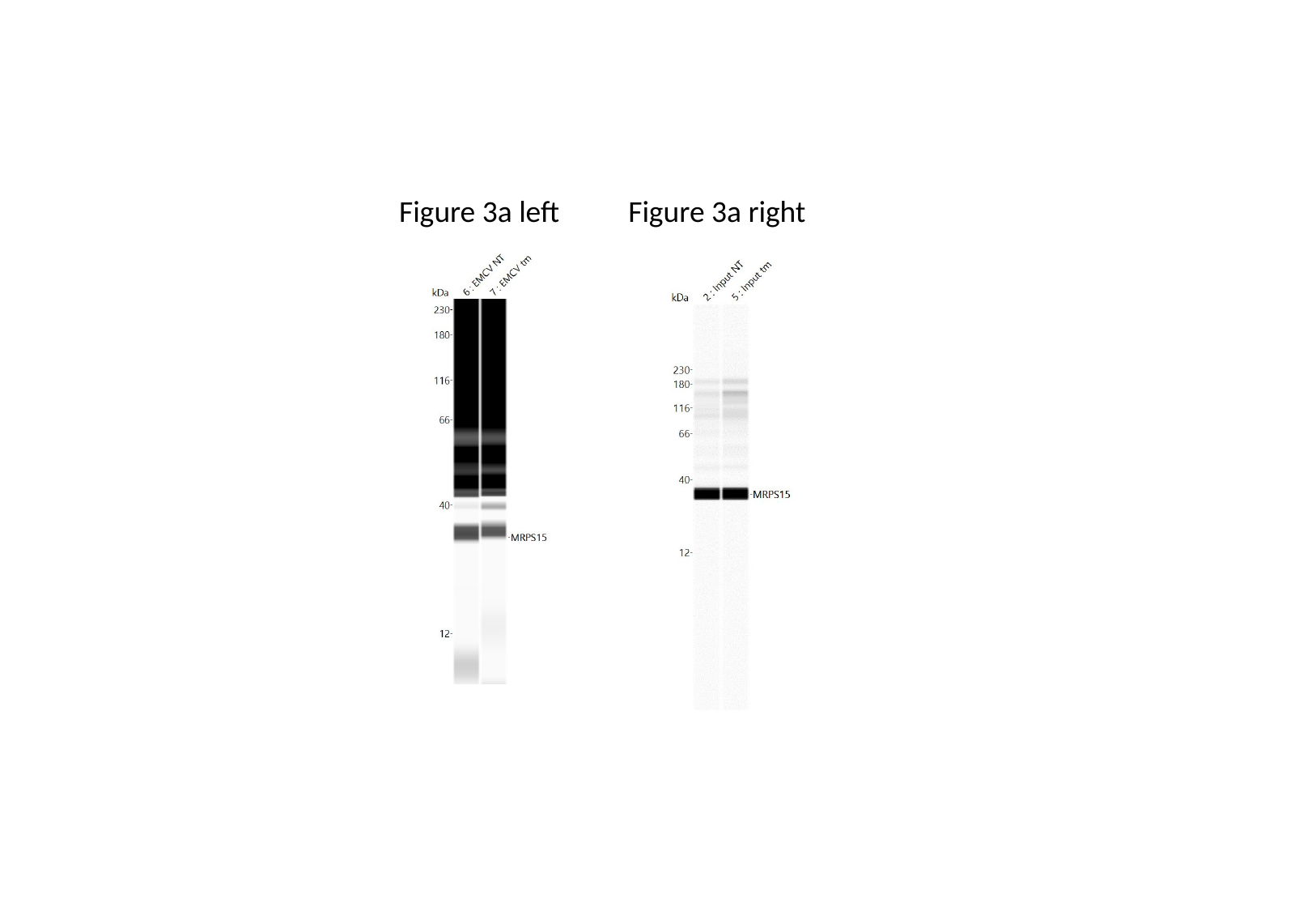

Figure 3a left
Figure 3a right

## Slide 2
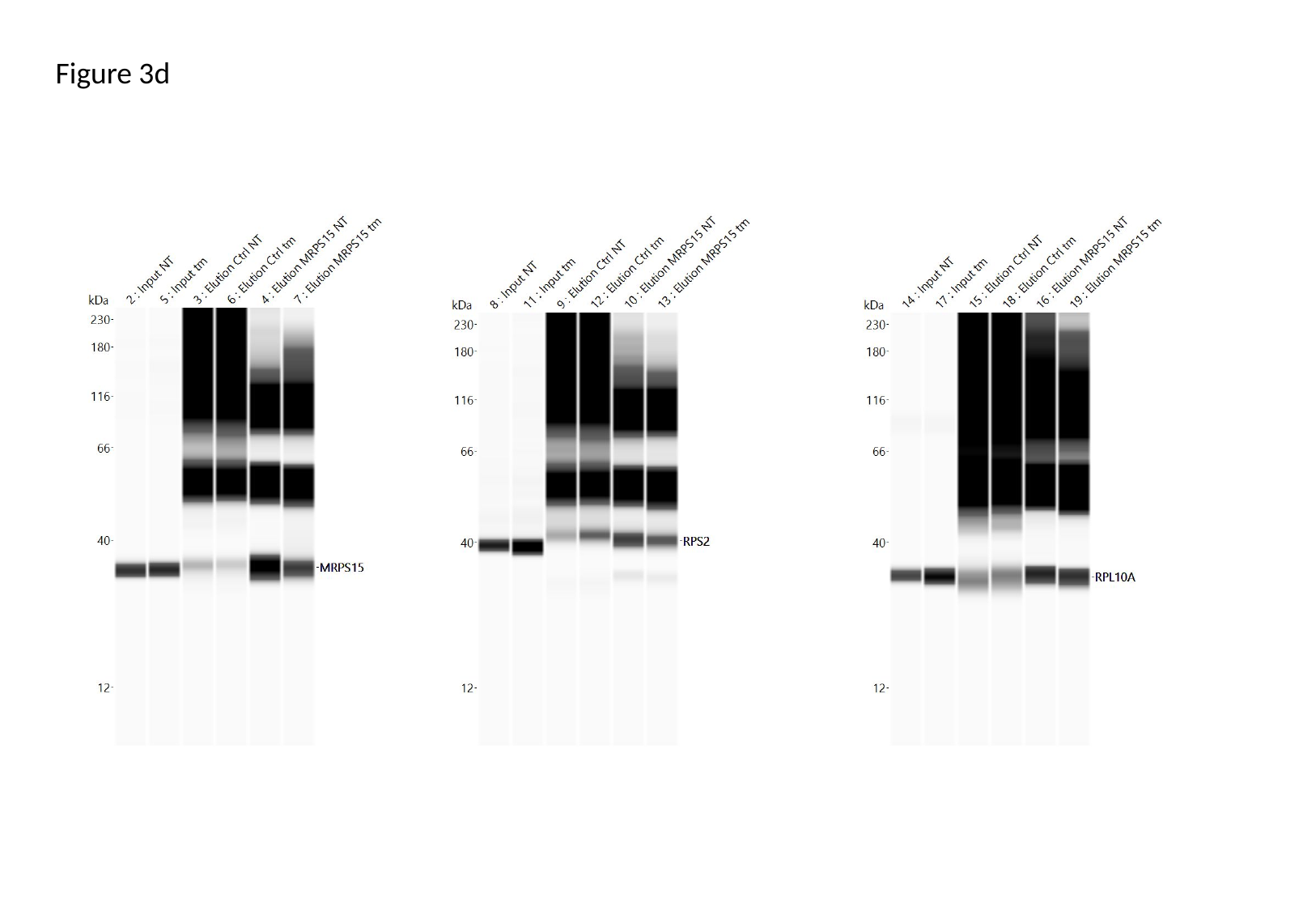

Figure 3d

## Slide 3
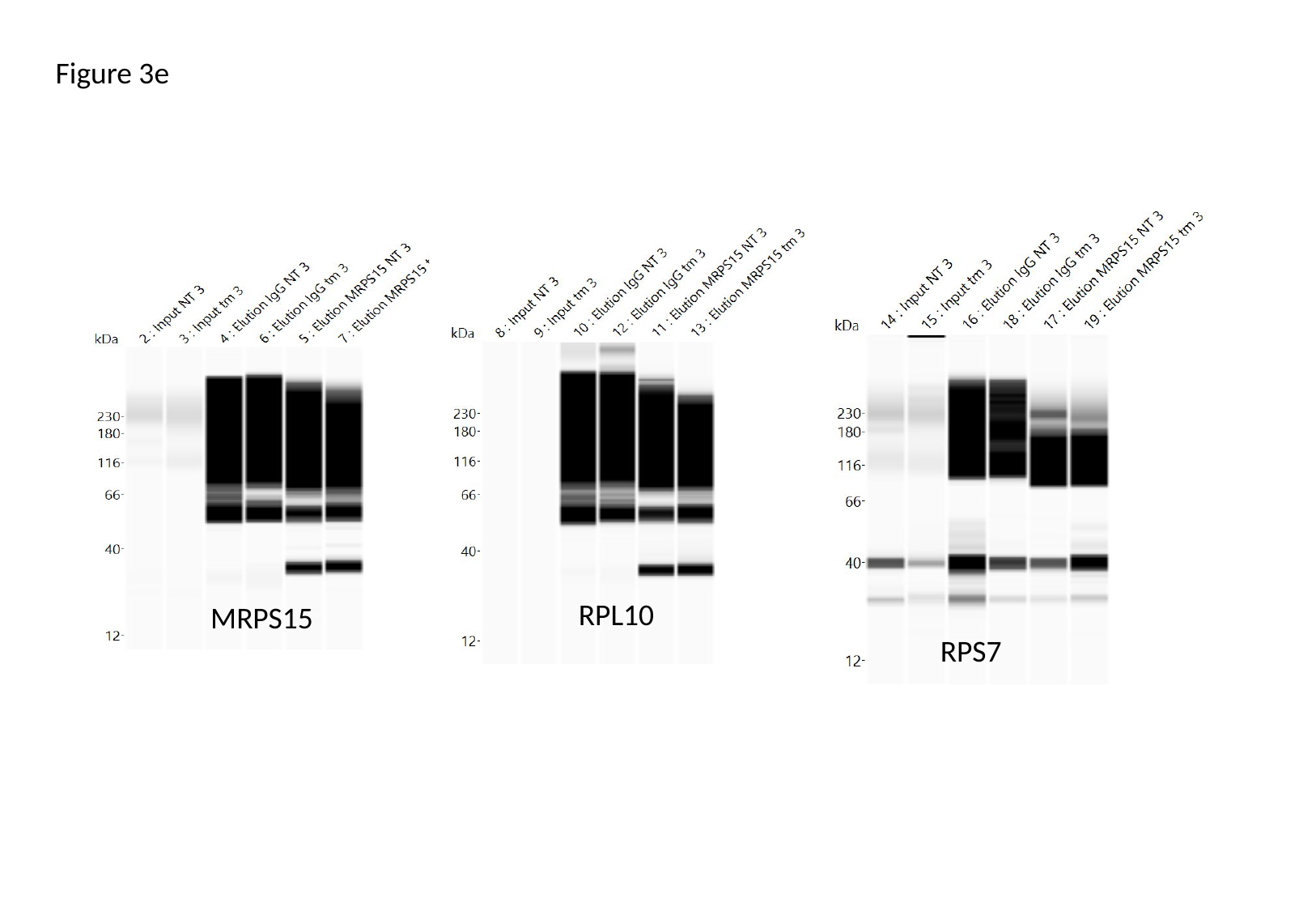

Figure 3e
RPS7
RPL10
MRPS15

Supplement: Supplementary file 1 [file ijms-25-03250-s001.zip › Supplementary Material/Figure 3 Full Blots (Jess).pptx]

## Slide 1
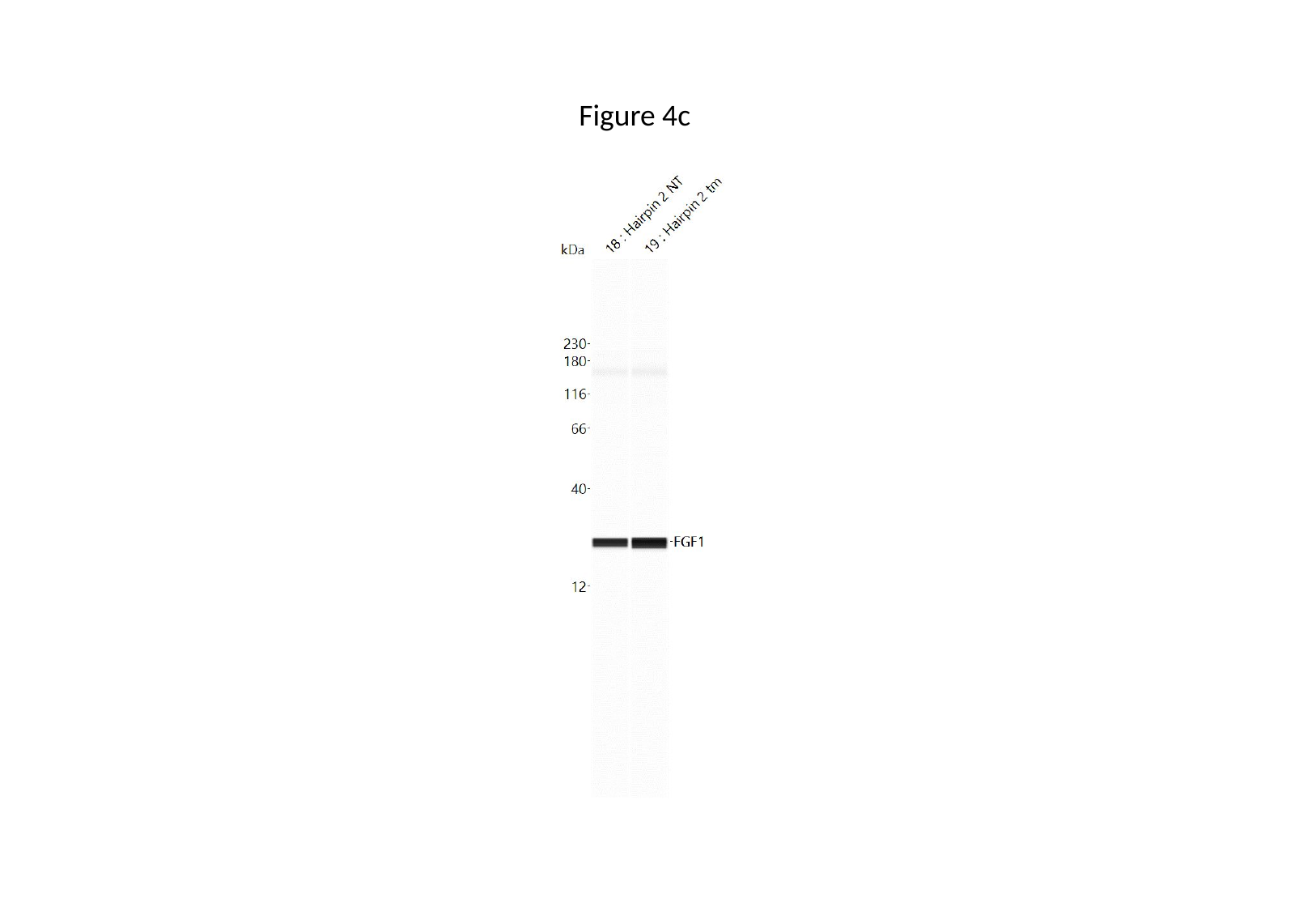

Figure 4c

Supplement: Supplementary file 1 [file ijms-25-03250-s001.zip › Supplementary Material/Figure 4 Full Blots (Jess).pptx]

## Slide 1
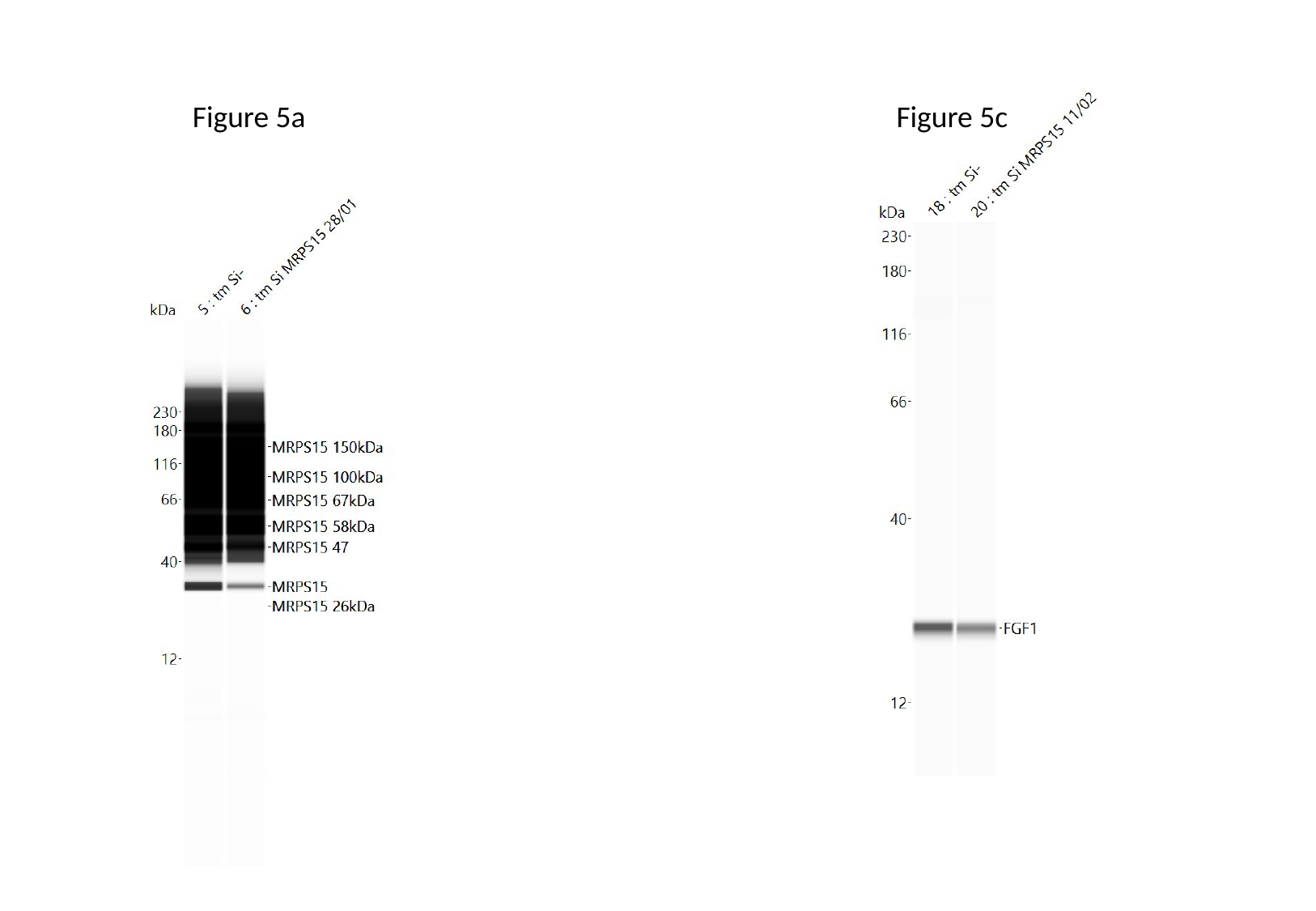

Figure 5a
Figure 5c

Supplement: Supplementary file 1 [file ijms-25-03250-s001.zip › Supplementary Material/Figure 5 Full Blots (Jess).pptx]

## Slide 1
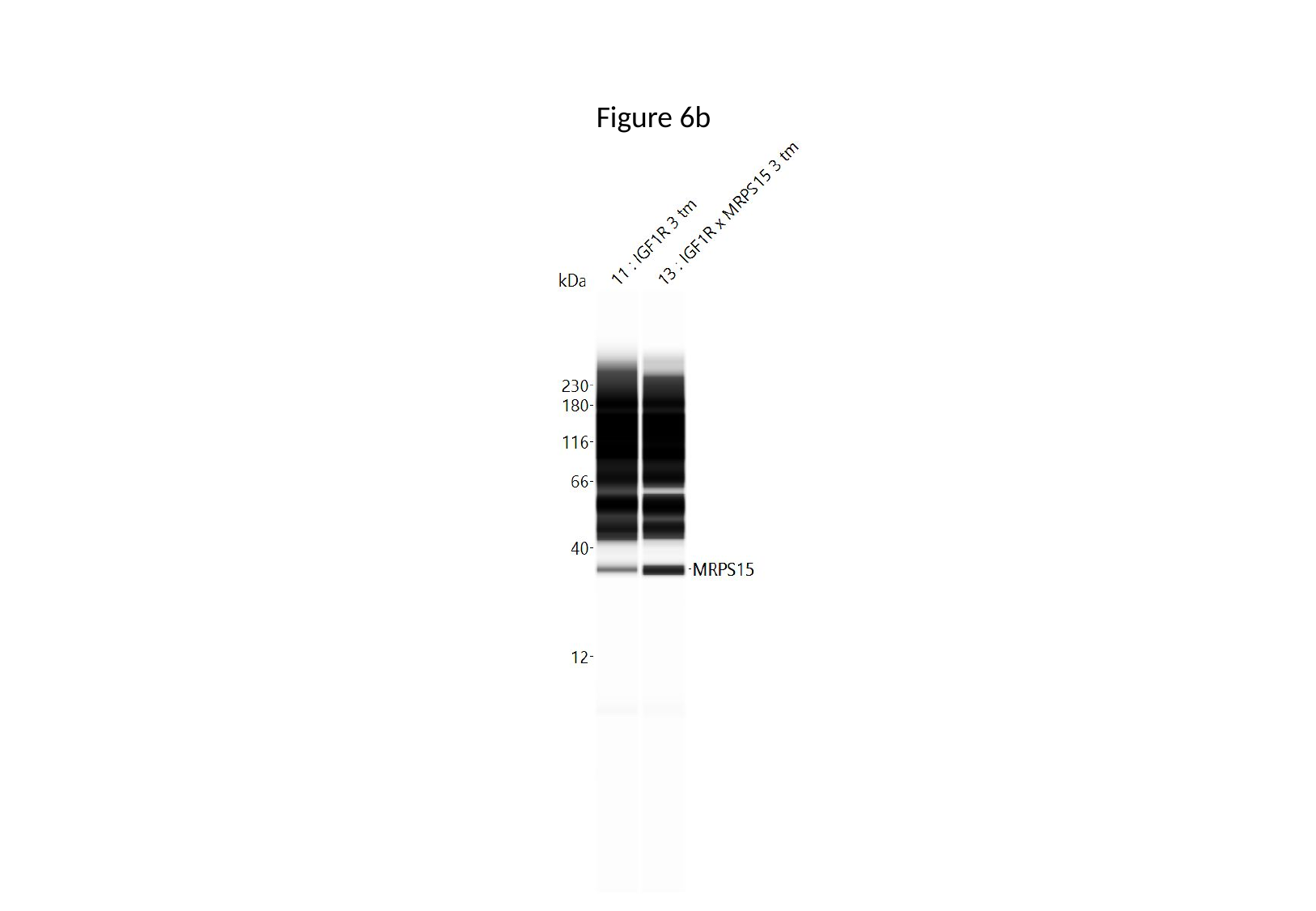

Figure 6b

Supplement: Supplementary file 1 [file ijms-25-03250-s001.zip › Supplementary Material/Figure 6 Full Blots (Jess).pptx]
